# Novel variants in *MLL* confer to bladder cancer recurrence identified by whole-exome sequencing

**DOI:** 10.18632/oncotarget.6380

**Published:** 2015-11-25

**Authors:** Song Wu, Zhao Yang, Rui Ye, Dan An, Chong Li, Yitian Wang, Yongqiang Wang, Yi Huang, Huan Liu, Feida Li, Luyun He, Da Sun, Yuan Yu, Qiaoling Li, Peide Huang, Meng Zhang, Xin Zhao, Tengteng Bi, Xuehan Zhuang, Liyan Zhang, Jingxiao Lu, Xiaojuan Sun, Fangjian Zhou, Chunxiao Liu, Guosheng Yang, Yong Hou, Zusen Fan, Zhiming Cai

**Affiliations:** ^1^ The Affiliated Luohu Hospital of Shenzhen University, Shenzhen Luohu Hospital Group, Shenzhen, China; ^2^ CAS Key Laboratory of Infection and Immunity, Institute of Biophysics, Chinese Academy of Sciences, Beijing, China; ^3^ CAS Key Laboratory of Pathogenic Microbiology and Immunology, Institute of Microbiology, Chinese Academy of Sciences, Beijing, China; ^4^ Department of Urological Surgery, Shenzhen Second People's Hospital, The First Affiliated Hospital of Shenzhen University, Shenzhen, China; ^5^ BGI-Shenzhen, Shenzhen, China; ^6^ Anhui Medical University, Hefei, China; ^7^ Department of Urology, Sun Yat-sen University Cancer Center, Guangzhou, China; ^8^ Department of Urology, Zhujiang Hospital of Southern Medical University, Guangzhou, China; ^9^ Guangdong Second People's Hospital, Guangzhou, China

**Keywords:** bladder cancer, tumor recurrence, MLL, drug-resistance, whole-exome sequencing

## Abstract

Bladder cancer (BC) is distinguished by high rate of recurrence after surgery, but the underlying mechanisms remain poorly understood. Here we performed the whole-exome sequencing of 37 BC individuals including 20 primary and 17 recurrent samples in which the primary and recurrent samples were not from the same patient. We uncovered that *MLL, EP400, PRDM2, ANK3* and *CHD5* exclusively altered in recurrent BCs. Specifically, the recurrent BCs and bladder cancer cells with *MLL* mutation displayed increased histone H3 tri-methyl K4 (H3K4me3) modification in tissue and cell levels and showed enhanced expression of GATA4 and ETS1 downstream. What's more, *MLL* mutated bladder cancer cells obtained with CRISPR/Cas9 showed increased ability of drug-resistance to epirubicin (a chemotherapy drug for bladder cancer) than wild type cells. Additionally, the BC patients with high expression of GATA4 and ETS1 significantly displayed shorter lifespan than patients with low expression. Our study provided an overview of the genetic basis of recrudescent bladder cancer and discovered that genetic alterations of *MLL* were involved in BC relapse. The increased modification of H3K4me3 and expression of GATA4 and ETS1 would be the promising targets for the diagnosis and therapy of relapsed bladder cancer.

## INTRODUCTION

Bladder cancer is the most common malignancy of urinary system after renal carcinoma worldwide. An approximately 430,000 new cases and an estimated 150,300 deaths per year [1, 2]. Urothelial bladder carcinoma (>90%) is the major type of bladder cancer and clinically divided into two major subtypes including non-muscle-invasive and muscle-invasive tumors [3]. About 70% of the urothelial bladder carcinoma patients are diagnosed with superficial non-muscle-invasive tumors which are apt to recur with the rate of 31% to 78% within five years but not life-threatening [4], while about 30% of the patients succumb to muscle-invasive tumors with a high risk of death from distant metastases [5]. Although transurethral resection, radical cystectomy, bricker operation combined with chemotherapy extend lifespan of BC patients, the problem of easy recurrence remains unsolved. It is very important to explicit the mechanisms of bladder cancer relapse.

A previous study indicated that 30.8% BC patients (135/438) developed intravesical recurrence with the median interval of 15 months in a median follow-up of 45 months in which lower tumor grade and tumor multifocality correlated with the risk of tumor relapse [6]. In another cohort of 1529 patients with primary superficial bladder cancer, the tumor size, number of tumors, the presence of carcinoma in situ, stage T1 and Grade 2-3 have been correlated with disease recurrence [7]. After transurethral resection of bladder cancer, intravesical instillation of Bacillus Calmette-Guerin (BCG) or mitomycin C decreased 30% and 15% of the recurrent rate of BC respectively [8].

In addition to clinicopathologic indicators, emerging results uncovered the potential molecular markers for bladder cancer recurrence. Among them, the expression of MDR1 increased in 89% of patients who showed recurrence resulting from the decreased methylation at the promoter region of MDR1 [9]. And high expression of VEGF appeared to be significantly associated with BC recurrence after nephroureterectomy [10]. Additionally, the expression of estrogen receptor (ER) positively correlated with recurrence free rate in non-muscle-invasive bladder cancer by suppressing cadherin switch [11]. Interestingly, epithelial mesenchymal transition (EMT) markers were indicated in the process of BC relapse [12, 13]. These studies supported that the gained expression of N-cadherin positively correlated with bladder carcinoma recurrence after nephroureterectomy.

Accumulating evidence suggests the relationship between the genetic alteration and BC recurrence. For example, the deletion of chromosome 9 was a predictive marker for tumor recrudescence [14]. Furthermore, *IL-6* gene promoter single nucleotide polymorphism (SNP) (G174C) was associated with increased recurrence risk in patients with maintenance BCG treatment and peroxisome proliferator-activated receptor-γ (PPARG) SNP (Pro12Ala) was associated with reduced recurrence risk in non-treated patients [15].

Although lots of markers have been reported as independent indicators for bladder cancer recurrence and prognosis, they are of limited applicable value. Firstly, the mRNA expression levels of MDR1 [9] and VEGFR [10] predicted a poor prognosis of bladder cancer patients, while the relationship of proteins expression levels of MDR1 and VEGFR and bladder cancer recurrence remain unclear. Furthermore, ER [11] and EMT markers [12, 13] were not promising markers of bladder cancer recurrence due to the subjectivity and false positivity of the immunohistochemistry method. Finally, the variant *IL-6* genotype was associated with an increased recurrence risk, its application scope should be examined in a larger cohort.

So far, the genetic analysis for recurrent BCs compared to primary ones is still at its preliminary stage. And no molecular target agents have been approved for the treatment of recurrent bladder cancer. Here we provide an insight into the difference of genetic basis between 17 recurrent BC samples and 20 primary ones with the method of exome sequencing and discover that the alterations of *MLL* are involved in BC relapse. *MLL* codes a transcriptional coactivator which plays an essential role in gene expression regulating during early embryo development and hematopoiesis [16]. It contains a SET (suppressor of variegation, enhancer of zeste, trithorax) domain at the C-terminus and belongs to a member of the MLL/trx family that specifically methylates lysine 4 on histone H3 (H3K4), a modification typically associated with transcriptionally active regions of chromatin [17]. Genomic rearrangements of the human chromosomal band 11q23 involving *MLL* are frequent events in pediatric leukemia, appearing in more than 70% of infant acute lymphoblastic leukemia (ALL) and approximately 10% of acute myelogenous leukemia (AML) cases [17]. Although MLL family members frequently altered in bladder cancer [5, 18, 19], the exact function of MLLs in bladder cancer remains unclear. In this paper, *MLL* particularly altered in recurrent bladder cancers with elevated modification of H3K4me3 and increased expression of GATA4 and ETS1 downstream. We introduced the specific mutations of *MLL* into bladder cancer cells with the method of CRISPR/Cas9 and the mutated cell exhibited enhanced H3K4me3 modification and elevated expression of GATA4 and ETS1 downstream, which endowed bladder cancer cells with the capability of drug-resistance to chemotherapy drug epirubicin. Taken together, *MLL* mutation, elevated GATA4 and EST1 would be the promising biomarkers for diagnosis and targets for treatment of bladder cancer recrudescence.

## RESULTS

### Frequently mutated genes identified in 37 bladder carcinomas

To improve our understanding of the genetic basis of recurrent bladder carcinoma, we performed whole-exome sequencing of 37 pairs of tumors and matched peripheral blood samples including 20 primary bladder tumors and 17 recrudescent ones parallelly in which the primary and recurrent samples were not from the same patient. (Supplementary Table 1). Then we acquired a mean coverage depth of ~60× for all the samples sequenced, with at least ~80% of the targeted bases being sufficiently covered (≥10×) (Supplementary Figure 1 and Supplementary Tables 2-3). Additionally, the average sequencing depth of these two groups remained similar and showed no significant difference (Supplementary Figure 1D). After several rigorous bioinformatic analysis steps (Online Methods), up to 4152 candidate somatic mutations and 831 insertions and deletions (indels) were identified in our 37 samples [20]. The C->T/G->A mutation dominated the mutation spectrum in 37 bladder cancer samples (Supplementary Figure 2). Moreover, the different types of single nucleotide variants (SNV) and indels displayed the similar mutation patterns between the primary and recurrent bladder cancer samples (Supplementary Figures. 3-4).

**Figure 1:**
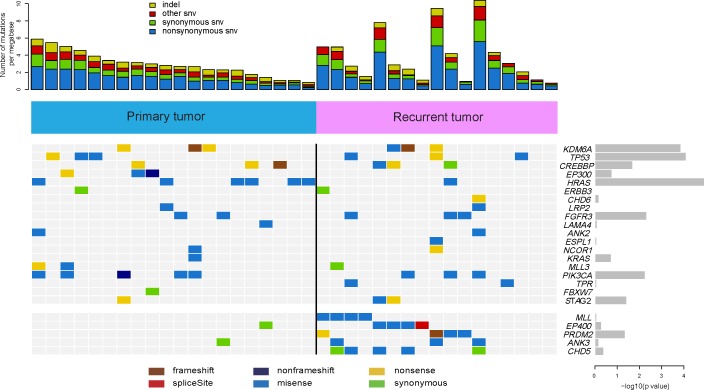
Frequently mutated genes identified in 37 bladder carcinomas Significantly mutated genes are listed on the right. The upper histogram shows the somatic mutation rate in 20 primary tumors and 17 recurrent tumors. The heat map in central shows the distribution of mutations across all samples. All the mutations shown were confirmed by Sanger sequencing.

In validation experiments, 1,023 (91%) somatic substitutions and 67 (74%) indels are confirmed respectively on 1,119 predicted somatic substitutions and 91 indels by Sanger sequencing20. In order to uncover the common and distinct altered genes between primary bladder cancer samples and recurrent ones, we assessed the statistical significance of the observed mutation prevalence for the genes with the method of mutcigCV [5]. In total, we identified 24 significantly mutated genes in 37 BCs (Figure 1 and Supplementary Table 4) including four well-known bladder cancer related genes (*TP53* [20, 21], *HRAS* [22], *FGFR3* [22], and *PIK3CA* [23, 24]).

Among the differentially mutated genes between the primary group and recurrent group samples, *MLL, EP400, PRDM2, ANK3* and *CHD5* mutations were identified and confirmed exclusively in the recurrent BCs (Figure 1 and Supplementary Table 4). Interestingly, three genes displayed significant difference between the two groups including *MLL* (encoding a transcriptional coactivator responsible for histone H3 lysine 4 (H3K4) methylation) [25], *EP400* (encoding a component of the NuA4 histone acetyltransferase complex that positively regulates transcription) [26] and *PRDM2* (encoding a nuclear histone/protein methyltransferase that regulates transcription during neuronal differentiation and tumorigenesis) [27] (Figure 1 and Supplementary Table 4). Taken together, the mutations of *MLL, EP400* and *PRDM2* may be involved in bladder carcinoma relapse.

### *MLL, EP400* and *PRDM2* exclusively alter in recurrent bladder carcinomas

**Figure 2:**
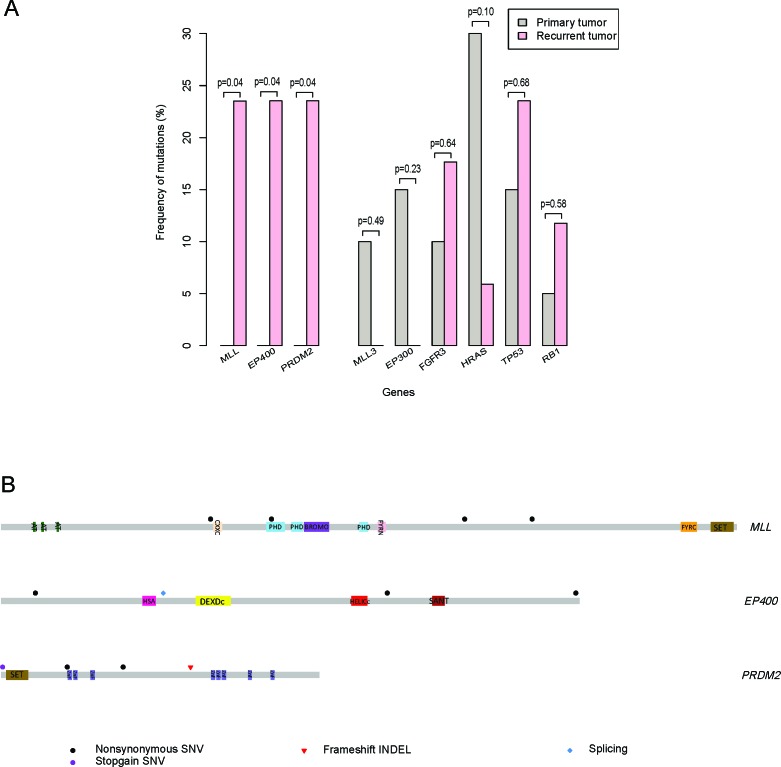
*MLL, EP400* and *PRDM2* exclusively alter in recurrent bladder carcinomas **A.** Mutation frequencies are expressed as a percentage of 20 primary (grey) and 17 recurrent (pink) samples. The *p*-value generated by fisher exact test is marked over bars. **B.** Somatic mutations in genes from recurrent samples.

To confirm the relationship between the specific gene mutation and the primary/recurrent tumors, mutation frequencies of *MLL, EP400, PRDM2, MLL3, EP300, FGFR3, HRAS, TP53* and *RB1* were calculated for differential analysis. In particular, *MLL, EP400* and *PRDM2* exclusively mutated in recurrent tumors (4/17, *p* = 0.04) which indicated their association with BC recrudescence. However, *MLL3* (2/20, *p* = 0.49) and *EP300* (3/20, *p* = 0.23) exclusively mutated in primary tumors indicating that chromatin remodeling genes (*MLL, MLL3, EP300* and *EP400*) may play distinct roles in primary and recurrent BC tumorigenesis (Figure 2A and Supplementary Table 5). In addition, non-muscle-invasive BC related genes *FGFR3* mutated in both primary tumors (2/20, 10.0%) and relapsed tumors (3/17, 17.6%) and *HRAS* altered in both primary tumors (6/20, 30.0%) and recurring tumors (1/17, 5.9%). But they showed no significant difference between these two groups (p=0.64 for *FGFR3* and *p* = 0.10 for *HRAS*) (Figure 2A). Similarly, muscle-invasive BC related genes *TP53* mutated in both primary tumors (3/20, 15.0%) and recurrent tumors (4/17, 23.5%) and *RB1* altered in primary tumors (1/20, 5.0%) and recrudescent tumors (2/17, 11.8%). But they displayed no remarkable difference between these two groups (*p* = 0.68 for *TP53* and *p* = 0.58 for *RB1*) (Figure 2A).

Then we focused on the exclusively mutated genes in recurrent BCs. In addition to the mutation frequencies of those genes, we analyzed the mutation status of *MLL, EP400* and *PRDM2*. In particular, 23.5% of the recurrent samples had *MLL* mutations (4 out of 17, *p* = 0.04), and all the mutations in *MLL* were non-synonymous SNV. Only one mutation named c.C4437G substitution located in the typical PHD domain and others located in the linker region instead (Figure 2B and Supplementary Table 4). Furthermore, 23.5% of the recurrent samples had *EP400* mutations (4 out of 17, *p* = 0.04) including one splicing named c.2629-1G>- and three non-synonymous SNV. However, none of the mutations located in the typical structural domain (Figure 2B and Supplementary Table 4). For *PRDM2*, 23.5% of the recurrent samples had *PRDM2* mutations (4 out of 17, *p* = 0.04) including one nonsense mutation, one frameshift-insertion and two non-synonymous SNV (Figure 2B and Supplementary Table 4). These results indicated that the specific mutation of *MLL* located in the PHD domain may influence the binding function of MLL and chromosome or targets. However, the nonsense and insertion mutations suggested that *PRDM2* may function as a suppressor in BC recurrence.

### *MLL* mutations in recurrent bladder carcinomas enhance the occupancy of H3K4me3 in *GATA4* and *ETS1* promoters

**Figure 3:**
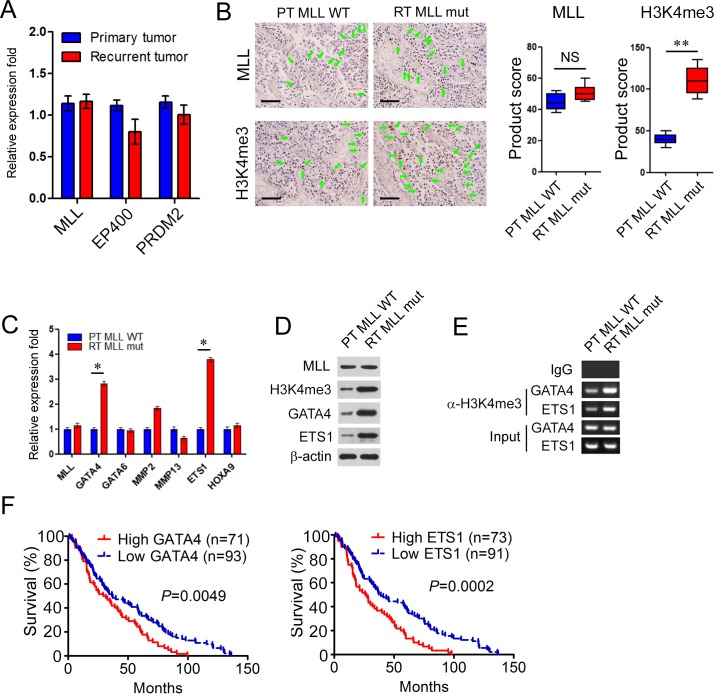
*MLL* mutation in recurrent bladder carcinomas enhance the occupancy of H3K4me3 in *GATA4* and *ETS1* promoters **A.** Real-time PCR analysis of MLL, EP400 and PRDM2 between the non-mutated primary group and the mutated recurrent group (*n* = 20 for primary tumors, *n* = 4 for recurring tumors). Data is displayed as mean ± SD. **B.** Representative immuno- histochemical staining of MLL in B234 and B112. Overall staining for MLL was measured by multiplication of staining percentage (0%-100%) and staining intensity on a numerical scale (none = 1, weak = 2, moderate = 3, strong = 4), resulting in an overall product score. Scale bar = 50 μm. **C.** Real-time PCR analysis of MLL, GATA4, GATA6, MMP2, MMP13, ETS1 and HOXA9 between the primary group with wild type MLL (PT MLL WT) and the recurrent group with mutated MLL (RT MLL mut) (*n* = 20 for primary tumors, *n* = 4 for recurring tumors). Data is expressed as mean±SD. **D.** Western blot analysis of MLL, GATA4 and ETS1 between PT MLL WT and RT MLL mut. β-actin was used as loading control. **E.** CHIP assay was performed using H3K4me3 antibody and IgG antibody in B234 and B112. **F.** Kaplan-Meier curves displayed survival rates of bladder carcinomas patients with high vs low expression levels of GATA4 or ETS1. n, patient number. *P < 0.05; **P < 0.01.

Accumulating studies indicate that the deregulation of remodeling complex and epigenetic regulators contribute to bladder carcinoma tumorigenesis [1, 5]. In our study, *MLL, EP400* and *PRDM2* exclusively mutated in the recurrent BCs. In order to uncover the mechanisms underlying BC recurrence mediated by these mutations, we first analyzed the mRNA expression levels of *MLL, EP400* and *PRDM2* by real time PCR (RT-PCR) between the non-mutated primary group and the mutated recurrent group (n=20 for primary tumors, n=4 for recurring tumors). The expression levels of *MLL, EP400* and *PRDM2* showed no significant difference between these two groups (Figure 3A). We next analyzed the levels of H3K4me3, acetylated histone H4 and H3K9me3 modification corresponding to the enzyme activity of *MLL, EP400* and *PRDM2* in the former two groups with the method of immunohistochemistry. Interestingly, only H3K4me3 modification did increase significantly (2.8 fold) in the recurrent tumors with *MLL* mutation (RT *MLL* mut, n=4) rather than that of primary tumor with WT *MLL* (PT MLL WT, n=20) with the same levels of MLL expression (Figure 3B). Overall staining for MLL and H3K4me3 was measured by multiplication of staining intensity on a numerical scale (none = 1, weak = 2, moderate = 3, strong = 4) and staining percentage (0%–100%), resulting in an overall product score (Figure 3B). However, the acetylated histone H4 and H3K9me3 modification displayed no significant difference between the respective paired groups (data not shown). These results suggested that *MLL* mutation may participate in bladder cancer recurrence dependent on the increased modification of H3K4me3.

As a histone methyltransferase, MLL catalyzes H3K4me3 modification promoting gene transcription [25]. To identify the target genes activated in *MLL* mutated recurrent group, some reported candidate targets (such as *GATA4, GATA6, MMP2, MMP13, ETS1* and *HOXA9*) related to tumorigenesis, drug resistance and cancer progression were selected [28]. In RT-PCR assay, the expression levels of GATA4 and ETS1 remarkably increased 2~3 fold in RT MLL mut than that of PT MLL WT (Figure 3C). To further confirm the up-regulation of GATA4 and ETS1 in *MLL* mutated tumors, tissue lysates were applied for western blot analysis. Similarly, MLL displayed same expression levels in RT *MLL* mut and PT *MLL* WT, but the modification of H3K4me3 and the expression of GATA4 and ETS1 significantly increased 4~5 fold in RT *MLL* mut than that of PT *MLL* WT (Figure 3D). We next carried out chromatin immunoprecipitation assay (ChIP) to investigate the H3K4me3 modification of *GATA* or *ETS1* promoters in former two groups. We observed a 5~6 fold enrichment of *GATA4* and *ETS1* promoters with the H3K4me3 antibody in RT *MLL* mut than that in PT *MLL* WT (Figure 3E). These results demonstrated that the H3K4me3 modification of *GATA* and *ETS1* promoters and the expression levels of GATA4 and ETS1 correlated with bladder cancer relapse.

Further in Suk-Chul Bae's datasets [29], the BC patients with high expression of GATA4 and ETS1 significantly displayed shorter lifespan than patients with low expression (*P* = 0.0049 for GATA4 and *P* = 0.0002 for ETS1) (Figure 3F). The elevated expression of GATA4 and ETS1 negatively correlated with the prognosis of bladder cancer patients and may be involved in bladder cancer relapse.

### Conformation of *MLL* mutations in a larger cohort

In this study, the 20 primary and 17 recurrent bladder cancer samples were not from the same patient. According to the clinical information of 37 bladder cancer samples, the stage ranged from Ta to T3 in the distinct two groups and the high grade samples composed 8/20 and 10/17 of primary and relapsed group respectively. Based on these two clinical characteristics, there is no significant difference between primary and recurrent groups. Nevertheless, in order to exclude the possibility that different *MLL* mutation frequencies resulted from different clinical characteristics, we validated the mutation status of *MLL* in a 40 low grade primary and 40 low grade relapsed bladder cancer samples, in which the stage ranged from Ta to T1 (Supplementary Table 6). The g.chr11:118359433C>G mutation from B112 was identified in three recurrent samples (SR14, SR29 and SR33), g.chr11:118374172C>T mutation from B73 was identified in two recurrent samples (SR17 and SR25), g.chr11:118375263C>G mutation from B71 was identified in SR5 recurrent sample, and g.chr11:118348796G>A mutation from W100 was identified in SR23 recurrent sample and all these alterations were not discovered in primary samples (Supplementary Table 6). Taken together, the specific mutations of *MLL* did exist in the recurrent bladder cancer samples, instead of the primary ones, which were not caused by different clinical characteristics between primary and recurrent groups.

### Bladder cancer cells with *MLL* mutation display decreased susceptibility to epirubicin *in vitro*

**Figure 4:**
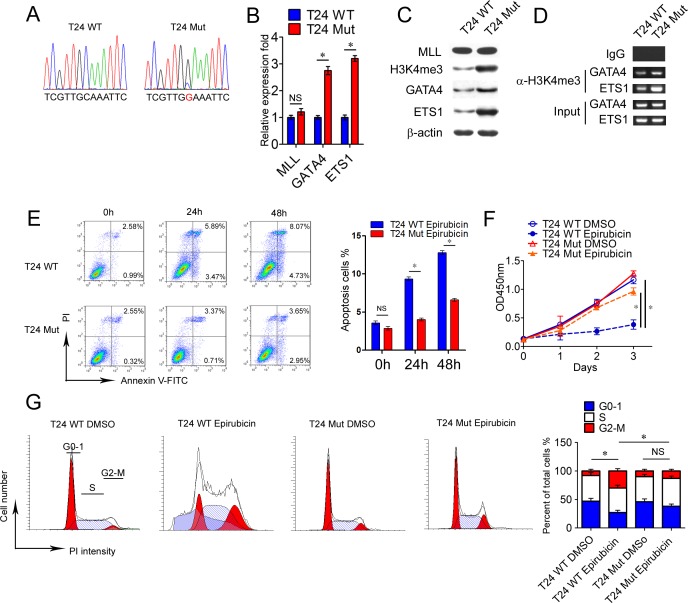
Bladder cancer cells with *MLL* mutation display decreased susceptibility to epirubicin **A.** Sanger sequencing of *MLL* PCR product in T24 WT and T24 Mut. **B.** Real-time PCR analysis of MLL, GATA4, and ETS1 mRNA levels in T24 WT and T24 Mut. Data is displayed as mean±SD. **C.** Western blot analysis of MLL, GATA4 and ETS1 in T24 WT and T24 Mut. β-actin was used as loading control. **D.** CHIP assay was performed using H3K4me3 antibody and IgG antibody in T24 WT and T24 Mut. **E.** Epirubicin was applied to induce apoptosis in T24 WT and T24 Mut and the cells were collected and analyzed at indicated point. Data is SSexpressed as mean±SD. **F.** The propagation curves of T24 WT and T24 Mut were measured by CCK8 with/without the treatment of epirubicin. Data is displayed as mean±SD. **G.** The cell cycle of T24 WT and T24 Mut were measured by PI staining with/without the treatment of epirubicin. Data is showed as mean±SD. *P < 0.05.

In order to illustrate the function of mutated *MLL* in bladder cancer cell lines, we selected c.C4437G substitution located in the PHD domain of *MLL* from sample B112 for the further functional experiments (Figure 2A and Supplementary Table 4). Then we screened this mutation in bladder cancer cell lines. However, EJ, T24, 5637 and BIU87 cell lines harbor the wild type sequence of *MLL* in chr11:118359433C position (hg19) (Figure 4A and data not shown). Then we applied the CRISPR/Cas9 technology [30] to obtain the specific mutation of *MLL* in T24 bladder cancer cell lines. After Sanger sequencing confirmation, we obtained the mutated T24 cells mimic the c.C4437G mutation (Figure 4A). At first, we compared the expression levels of MLL between T24 wild type cells (T24 WT) and T24 mutation cells (T24 Mut) and found that the quantity of MLL remained unchanged in T24 Mut in both mRNA and protein levels compared with T24 WT (Figure 4B and 4C). Nevertheless, the H3K4me3 modification increased 4 fold in T24 Mut than that of T24 WT (Figure 4C). Meanwhile, GATA4 and ETS1 as MLL targets increased their expression 2~3 fold in T24 Mut than that of T24 WT (Figure 4B and 4C). Additionally, 2~3 fold and 5~6 fold H3K4me3 modification harbored the promoters of *GATA4* and *ETS1* respectively in T24 Mut than that of T24 WT (Figure 4D). These data demonstrated that the specific mutation of *MLL* in T24 bladder cancer cells promoted the transcription of *GATA4* and *ETS1*.

To further verify the drug-resistant function of *MLL* mutation in bladder cancer cells, epirubicin was used in the functional experiments *in vitro*, which is widely used as a chemotherapeutic drug in intravesical instillation for bladder cancer patients [31]. 10 μg/ml epirubicin was applied to induce apoptosis of T24 WT and T24 Mut for 24h and 48h. Results from fluorescence-activated cell sorting (FACS) indicated that T24 WT showed 2 fold Annexin-V positive apoptosis cells than that of T24 Mut after epirubicin treatment for 24h and 48h (Figure 4E). To exam whether *MLL* mutation influenced cancer cell proliferation and cell cycle progression, T24 WT and T24 Mut showed comparable proliferation rate without epirubicin in the proliferation assay (Figure 4F). Under the stimulation of epirubicin, T24 WT displayed significant decrease in cell numbers (~30%) while T24 Mut kept a relative high rate of proliferation (Figure 4F). Similar results obtained from cell cycle analysis, T24 WT and T24 Mut displayed homologous cell cycle distribution without epirubicin. Epirubicin treatment decreased the ratio of G0-1 phase (58%) and induced cell cycle arrest in G2-M phase in T24 WT (375%), but T24 Mut showed no obvious change (Figure 4G). These results illustrated that *MLL* mutation had no intrinsic influence on the propagation ability and cell cycle progression, but changed the susceptibility of bladder cancer cells to chemotherapeutics.

Actually, the acquisition of the rest of the three mutations of *MLL* were carried out. Unfortunately, there is no target sites recognized by *Streptococcus pyogenes Cas9* (PAM, a protospacer adjacent motif) around the mutation site g.chr11:118375263C>G from B71, and this mutation could not be obtained by the method of Cas9 described in this study. Moreover, the cell line with the mutation of g.chr11: 118348796G>A from W100 were not screened through three independent experiments. Finally, we got the T24 cell line with the mutation of g.chr11:118374172C>T from B73 (T24 Mut2) with the same method (Supplementary Figure 6A). In a similar manner, the mutation of *MLL* had no effect on the expression itself (Supplementary Figure 6B and 6C), but activated the transcription of targets *GATA4* and *ETS1* (Supplementary Figure 6B-6D). Additionally, although T24 Mut2 cells displayed analogous proliferation ability, they harbored the elevated drug-resistance capability to epirubicin and showed less apoptosis cells (42%), more cells survival (329%) and normal cell cycle distribution compared with T24 WT cells with the treatment of epirubicin (Supplementary Figure 6E-6G). Taken together, GATA4 and ETS1 overexpression mediated by *MLL* mutation enhanced the ability of drug-resistant to epirubicin which endowed mutated bladder tumor cells the advantage of survival and recurrence in the presence of chemotherapeutics.

### GATA4 and ETS1 participate in the drug-resistance of *MLL* mutated bladder cancer cells

**Figure 5:**
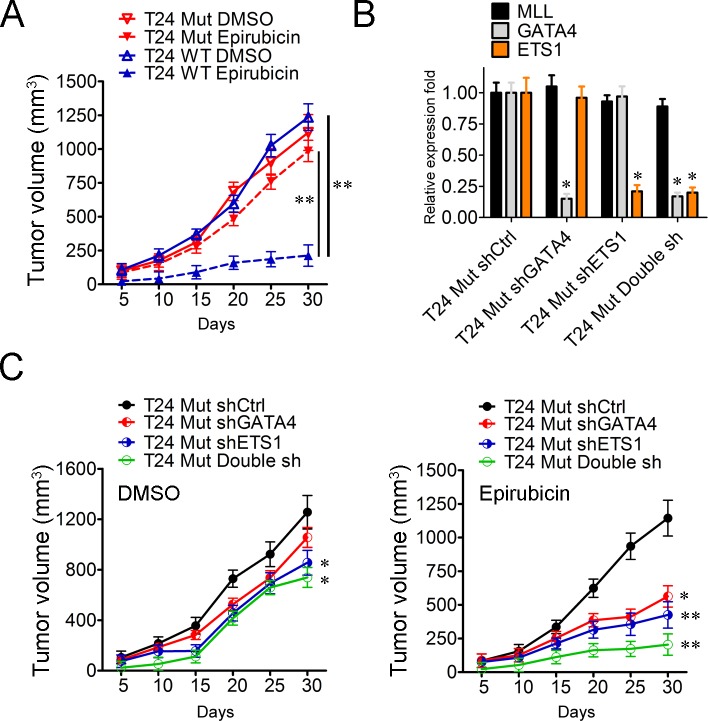
GATA4 and ETS1 participate in the drug-resistance of *MLL* mutated bladder cancer cells **A.** Tumor formation assays of T24 WT and T24 Mut cells with DMSO or epirubicin. The volume of xenografts was measured every five days, V = (π/6)×abc. *N* = 5, data is displayed as mean ± SD. **B.** Real-time PCR analysis of GATA4 and ETS1 in T24 Mut shCtrl, T24 Mut shGATA4, T24 Mut shETS1 and T24 Mut Double sh. These results were repeated for three times. Data is displayed as mean ± S **C.** Subcutaneous tumor model T24 Mut shCtrl, T24 Mut shGATA4, T24 Mut shETS1 and T24 Mut Double sh with DMSO or epirubicin. Five days later, the mice were grouped and administered intraperitoneally with DMSO or epirubicin at a dose of 2 mg/kg two times per week for 30 days. The volume of xenografts was measured every five days. *N* = 5, data is displayed as mean ± SD. *P < 0.05; **P < 0.01.

In order to clarify the function of *MLL* mutation *in vivo*, xenograft bladder cancer models in mice were thus employed in which 2×10^6^ T24 WT and T24 Mut cells were injected subcutaneously into the back of nude mice respectively. One week after tumor implantation, 2 mg/kg epirubicin or DMSO was injected intraperitoneally twice per week and the volume of established tumors was measured every five days. On day 30, the mice were sacrificed and the therapeutic effect was evaluated. With the addition of DMSO, T24 WT and T24 Mut displayed analogous propagation curves (Figure 5A). While under the treatment of epirubicin, the proliferation of T24 WT was significantly suppressed (17.23%, *P* < 0.01), but T24 Mut cells could still establish the remarkable tumors comparable to that in DMSO condition (87.80%, *P* > 0.05) (Figure 5A). Moreover, the tumor formation of T24 WT and T24 Mut under the treatment of epirubicin showed significant difference (*P* < 0.01) (Figure 5A). These data indicated that, in consistence with the data from *in vitro, MLL* mutation enhanced the ability of drug-resistance in bladder cancer *in vivo*. As the activated targets in *MLL* mutation cells, whether GATA4 and ETS1 participated in this drug-resistance process was also examined *in vivo*. In the first place, GATA4, ETS1 and both of them were silenced with lentivirus and the efficiencies of knockdown were examined in RT-PCR experiments (Figure 5B). Then the four types of cells including T24 Mut shCtrl, T24 Mut shGATA4, T24 Mut shETS1 and T24 Mut Double sh were carried out in xenograft bladder cancer models in mice. Under the addition of DMSO, T24 Mut shETS1 and T24 Mut Double sh displayed a remarkable decrease in tumor volume, which suggested that the depletion of ETS1 (31.85%, *P* < 0.05) and both of GATA4 and ETS1 (41.16%, *P* < 0.05) inhibited the growth of T24 Mut cells (Figure 5C). Finally, the tumor formations of T24 Mut shGATA4 (50.83%, *P* < 0.05), T24 Mut shETS1 (62.79%, *P* < 0.01) and T24 Mut Double sh (82.10%, *P* < 0.01) were significantly suppressed compared with T24 Mut shCtrl with the addition of epirubicin (Figure 5C). Collectively, T24 Mut cells showed robust cell proliferation and tumor formation even under the treatment of epirubicin compared to T24 WT cells. When *GATA4* and *ETS1* were depleted, the tumor propagation and formation were significantly inhibited. All these data indicated GATA4 and ETS1 played essential roles in the drug resistance of *MLL* mutated bladder cancer cells.

## DISCUSSION

The recurrence of bladder carcinoma (BC) is the key problem need to be solved for both clinicians and scientific researchers, while none effective prevention and treatment strategies have been proposed during last thirty years. In this study, 20 primary and 17 recurrent bladder cancer samples were collected for whole-exome sequencing. After discovery and confirmation, five genes *MLL, EP400, PRDM2, ANK3* and *CHD5* significantly correlated with the recurrence of bladder cancer. Specifically, *MLL* mutation in recurrent bladder cancer samples and T24 cell lines acquired the elevated H3K4me3 activity and transcriptional activation of target genes *GATA4* and *ETS1*. The increased expression of GATA4 and ETS1 contributed to enhance the drug-resistance ability during the progression and recurrence of bladder cancer. Nevertheless, the primary and recurrent samples applied in exome sequencing were not from the same patient, the mutation status and distinct functions of *MLL, EP400, PRDM2, ANK3* and *CHD5* should be better researched and confirmed in primary and recurrent samples obtained from the same patient in the future study. It will reveal whether the specific mutation plays an indispensable role in bladder cancer recrudescence.

In the former studies, several candidate markers were indicated in the process of BC recurrence. For example, high expression of MDR1 [9], VEGF [10] and NCAD [12, 13] significantly associated with BC recurrence after nephroureterectomy. In addition, the expression of estrogen receptor (ER) positively correlated with recurrence free rate in non-muscle-invasive bladder cancer by suppressing cadherin switch [11]. With DNA methylation array analysis, Kim YJ et, al discovered that the highly methylation pattern in promoters of HOXA9, ISL1 and ALDH1A3 and their decreased protein expression levels significantly associated with aggressive clinicopathologic features [32]. Moreover, the deletion of chromosome 9 and the single nucleotide polymorphism (SNP) of *IL-6* gene promoter (G174C) positively correlated with BC relapse [15]. However, the SNP of PPARG (Pro12Ala) was negatively associated with the recurrent BC [15]. Although several molecular markers of bladder cancer prognosis have been identified, the limited value of current prognostic markers has created the need for new molecular indicators of bladder cancer progression and recurrence.

Unfortunately, all these markers or targets above were not applied into the clinical diagnosis and treatment yet. In order to uncover the genetic basis of recurrent BC compared to primary ones for the better understanding of bladder cancer recurrence and searching for the targets for its therapy. We carried out the whole-exome sequencing of recurrent and primary bladder cancer samples parallelly. Beyond confirming mutations in genes previously identified in BC, we uncovered five frequently altered genes exclusively in recurrent BC including *MLL, EP400, PRDM2, ANK3* and *CHD5*. In particular, four recurrent BCs harbored four distinct non-synonymous SNV in *MLL* locus in which one mutation located in the typical PHD domain named c.C4437G substitution.

*MLL* usually underwent frameshift mutation and rearrangement in bladder carcinoma and leukemia, in which *MLL* lost its normal function including the methyltransferase activity. On the one hand, the knockout of *MLL* in normal bladder epithelium would promote the initiation of bladder cancer according to the data from high throughput sequencing, in which three out of nine is nonsense or indel mutation [5], two out of nine is indel mutation [20] and more than 50% mutations were nonsense [1]. It means that approximately 20%~50% bladder cancer initiations positively correlated with the nonsense or loss-of-function of *MLL*. Thus, combined with others carcinogenic factors, the knockout of *MLL* in normal bladder epithelium could induce the tumorigenesis of bladder cancer. On the other hand, if *MLL* played as also a tumor suppressor in the formative bladder cancer hierarchy, it is could be speculated the knockout of *MLL* may help the bladder cancer cells into a more aggressive type, which should be further investigated. However, the same protein in the different stage of tumor formation may function in the distinct ways, at least in the current study. In detail, four specific mutations of *MLL* (g.chr11:118359433C>G, B112; g.chr11:118375263C>G, B71; g.chr11:118375263C>G, B73 and g.chr11: 118348796G>A, W100) exclusively occurred in recurrent bladder cancer samples rather than the primary ones and these mutations belonged to the gain-of-function, different from that of loss-of-function in primary bladder cancers.

In our study, the c.C4437G substitution in PHD domain and c.C7565T mutation of *MLL* locus do not change the mRNA and protein expression levels but increased the whole level of H3K4me3 modification in the recurrent group than that of primary group. According to the previous study, Wang P et, al. undertook a genome-wide analysis of H3K4 methylation patterns in Mll1(^+/+^) and Mll1(^−/−^) mouse embryonic fibroblasts (MEFs) [28]. We selected six candidates related to tumorigenesis, drug-resistance and cancer progression, and discovered that the promoters of *GATA4* and *ETS1* were occupied with more H3K4me3 modification resulting in enhanced expression of mRNA and protein levels of GATA4 and ETS1 in recurrent tumors and T24 Mut with *MLL* mutation compared with primary tumors and T24 WT with wild type *MLL* respectively.

*GATA4* belongs to a member of the GATA family of zinc-finger transcription factors which is indicated in the process of derivation and propagation of urothelium from hESCs and hiPS cells normally [33]. Besides the normal development function, *GATA4* was reported as a potential tumor suppressor of colorectal cancer, lung cancer and ovarian cancer [34-36]. Moreover, recent studies indicated an oncogene function of *GATA4* in cancer, including gastric carcinoma, breast carcinoma and ovarian cancer [37-39]. However, the relationship between *GATA4* and bladder cancer have not been reported so far. *ETS1* encodes a member of the ETS family of transcription factor which is involved in stem cell development, cell senescence and death, and tumorigenesis. Sari, Aysegul et al. found that non-muscle-invasive tumors had significantly higher ETS1 expression than that of the muscle-invasive tumors [40], indicating its special role in superficial bladder carcinoma. Additionally, the promoter of telomerase reverse transcriptase (*TERT)* gene frequently mutated in bladder carcinoma in which ETS1 bond to the altered promoter and promoted bladder carcinoma migration and progression [41].

In this study, we introduced the *MLL* mutation into bladder cancer transitional cell line T24 and confirmed that the mutation didn't influence the expression of MLL but increased the H3K4me3 modification in *GATA4* and *ETS1* promoters and the expression levels of GATA4 and ETS1. Furthermore, epirubicin induced wild type T24 cells undergoing obvious apoptosis and inhibited cell propagation significantly *in vitro*. However, T24 Mut displayed slightly increased apoptosis and kept the rapid proliferation rate under the stimulation of epirubicin. Additionally, T24 WT and T24 Mut displayed homologous cell cycle distribution in cell cycle analysis without epirubicin. Epirubicin treatment decreased the ratio of G0-1 phase and induced cell cycle arrest in G2-M phase in T24 WT, but T24 Mut showed no obvious change. All these data supported that *MLL* mutation in recurrent tumors and T24 cells enhanced the drug-resistant ability. As for their mutation types and protein functions, we speculated that these three mutations belonged to gain-of-function mutations and the altered the protein harbored the enhanced activity of H3K4me3 modification in target genes such as *GATA4* and *ETS1* dependent on the data from primary tissues (Figure 3) and cell lines with the specific mutations (Figure 4). Moreover, GATA4 and ETS1 played an indispensable role in the drug-resistance of bladder cancer cells with *MLL* mutation (Figure 5).

Nevertheless, these nonsynonymous mutations of bladder cancer are different from those alterations from acute lymphoid leukemia and acute myeloid leukemia in which the translocation and rearrangement dominate the mutation types. From the aspect of results of these two distinct mutation types, the gain-of-function of these bladder cancer specific mutations activating *GATA4* and *ETS1* seem like the translocation and rearrangement alterations from leukemia in which the *MLL* truncation and fusion proteins activated the transcription of target genes such as *Hox* genes and induced cell transformation.

Taken together, in recurrent bladder cancer samples with *MLL* alterations, the mRNA and protein expression levels of MLL displayed unchanged, but the H3K4me3 enzyme activity of MLL increased significantly. Meanwhile, the target genes *GATA4* and *ETS1* were activated through the elevated H3K4me3 modifications in their promoters which were mediated by the methyltransferase MLL. These results indicated that the mutated MLL protein may have a distinct structure which contributes to the close binding between MLL and other cofactors or DNA sequence, resulting in a swifter recruitment of other cofactors, DNA binding and H3K4me3 modifications in specific targets, such as *GATA4* and *ETS1* in this study. Furthermore, the enhanced expression of GATA4 and ETS1 indeed contributed to the drug-resistance of bladder cancer cells. Thus, *MLL* mutation, GATA4 and ETS1 may be used as a the biomarkers for diagnosis and targets for treatment of bladder cancer recrudescence.

Nevertheless, the expression profile of *MLL* mutated tumor cells and the mechanism of drug-resistant mediated by transcription factors GATA4 and ETS1 should be further identified. Additionally, the contribution of specific alterations of *EP400, PRDM2, ANK3* and *CHD5* to bladder carcinoma recurrence should also be further investigated.

## MATERIALS AND METHODS

### Genomic DNA extraction and Illumina based whole-exome sequencing

Genomic DNAs of tumor and matched peripheral blood samples from 37 bladder carcinoma patients were isolated and the DNA libraries were constructed according to the protocol provided by the manufacturer. For whole-exome sequencing (WES), genomic DNAs from the same 37 tumor-blood pairs were fragmented and subjected to whole-exome captured by SureSelect Human All Exon 50Mb Kit (Agilent Technologies). Then the libraries were pair-end sequenced on HiSeq 2000 platform to generate 2×100 bps reads. The insert size of the sequence library was 200bp ~ 300bp.

### Read mapping and detection of somatic mutations

Exact same pipeline was used as previously described [20]. After removing reads containing sequencing adaptors and low-quality reads with more than five unknown bases, the high quality reads were gapped aligned to the NCBI human reference genome (hg19) using BWA. Then, we performed local realignment of the BWA aligned reads and base quality recalibration using the Genome Analysis Toolkit (GATK) (1.2-44-g794f275). The raw lists of potential somatic substitutions were called by VarScan (v2.2.5). In this process, several heuristic rules were applied: (i) both the tumors and matched normal samples should be covered sufficiently (≥ 10×) at the genomic position being compared; (ii) the average base quality for a given genomic position should be at least 15 in both the tumors and normal samples; (iii) the variants should be supported by at least 10% of the total reads in the tumors while no high quality variant-supporting reads are allowed in normal controls; (iv) the variants should be supported by at least five reads in the tumors.

Using the same criteria, the preliminary lists of somatic indels was called out by GATK based on the local realignment results. After these two steps, germline variants could be effectively removed. To further reduce the false positive calls, variations including single nucleotide variants (SNVs) and indels were called with the SAMtools software package in the tumors. We eliminated all somatic variants that fulfill any one of the following filtering criterion: (i) variants with Phred-like scaled consensus scores or SNP qualities < 20; (ii) variants with mapping qualities < 30; (iii) indels represented by only one DNA strand; (iv) substitutions located 30bp around predicted indels. To deal with false positives associated with pseudo gene issues or repeat sequences, simulated reads (80bp in length) containing the potential mutations were generated and aligned to the reference genome. For a given variants, if more than 10% of the simulated variant-containing reads could not be uniquely mapped to the reference genome, this variant would be discarded. In order to eliminate any previously described germline variants, the somatic mutations were cross-referenced against the dbSNP (version 137). Any mutations present in above data sets were filtered out and the remaining mutations were subjected to subsequent analyses. In these two process, MutSigCV_1.4 was used for identification of the significantly mutated genes.

### Validation of somatic substitutions and indels by Sanger sequencing

Non-silent somatic substitutions and indels were validated by Sanger sequencing. The PCR primers for putative somatic variants were designed by primer 3 in silicon and initially used to amplify the source DNA from the tumors. PCR was performed on Dual 96 well GeneAmp PCR System 9700 (Applied Biosystems) and 20ng template DNA from each sample was used per reaction. The products were sequenced by 3730xl DNA Analyzer (Applied Biosystems). All sequences were analyzed by the Sequencing Analysis Software Version 5.2 (Applied Biosystems). To determine the somatic status of the confirmed mutation in tumor, the same primer pair was used to amplify the matched blood DNA.

The *MLL* mutations discovered exclusively in recurrent bladder tumors have been validated in a larger cohort (Supplementary Table 6). The primer pairs for *MLL* amplification are included in Supplementary Table 7.

### The significantly mutated genes

MutsigCV was used to prioritize the significantly mutated genes in primary and recurrent bladder carcinomas respectively.

### Primary antibodies

The primary antibodies were rabbit polyclonal to GATA4 (Abcam, US; ab84593), rabbit polyclonal to ETS1 (Abcam, US; ab26096), rabbit polyclonal to Histone H3 (trimethyl K4)-ChIP Grade (Abcam, US; ab8580), mouse anti-human MLL antibody (Santa Cruz Biotechnology, US; sc-374392) and mouse Anti-β-actin antibody (Sigma, Germany; A1978). Corresponding species-specific horseradish-peroxidase (HRP) labelled secondary antibodies (Pierce, US) were used.

### Western blot

Tumor tissues were cut into pieces and lysed with RIPA buffer [42] (50 mM Tris-HCl, 150 mM NaCl, 0.5% sodium desoxycholate, 0.1% SDS, 5 mM EDTA, 2 mM PMSF, 20 mg/ml aprotinin, 20 mg/ml leupeptin, 10 mg/ml pepstatin A, 150 mM benzamidine, and 1% Nonidet P-40) for 30min on ice, followed by centrifugalization, supernatant was collected for protein quantification. A total of 40μg of protein was added in a 4–12% gel and probed with according antibodies as previously described43.

### Real-time polymerase chain reaction (RT-PCR)

Total RNA was isolated from bladder carcinomas and human bladder cancer cell lines using the RNA isolation kit (Tiangen Biotech, CHN) according to the manufacturer's protocol. RNA was then subjected to cDNA synthesis using M-MLV Reverse Transcriptase (Promega, US) according to the manufacturer's protocol. The cDNA was then processed for an amplification step with the SYBR Green reaction system (Tiangen Biotech, CHN) running on an ABI 7300 machine (Applied Biosystems, US). The RT-PCR results were normalized using β-actin as endogenous control.

### Cell and sample description and preparation

The Cell line T24 was obtained from American Type Culture Collection (Rockville, MD, US). The primary and recurrent tumor samples with matched peripheral blood were obtained from bladder carcinoma patients newly diagnosed at the member institutions of the Urinogenital Cancer Genomics Consortium (BCGC) in China with informed consent. Detailed clinical information for the patients is summarized in Supplementary Tables 1 and 6. All the specimens were snap-frozen in liquid nitrogen upon collection and immediately stored at −80°C for further study. Especially, the primary tumor samples were transurethral resection specimen taken prior to the administration of any chemotherapy, whereas the recurrent tumor samples were all transurethral resection specimens taken from patients who had received epirubicin chemotherapy after the former transurethral resection. Clinically, an immediate instillation of chemotherapy after transurethral resection of bladder tumor (TURBT) or extended partial cystectomy was given to all patients (50mg epirubicin in 50ml saline). All patients were followed up for 3 years with a cystoscopy per 3 months. The recrudescent tumor samples from nine patients with localized muscle-invasive bladder carcinoma who underwent extended partial cystectomy resulted from their refusal of radical cystectomy strongly or their vital status limits.

### Cell proliferation assay

The proliferation and viability of T24 WT and T24 Mut/T24 Mut2 were measured by the cell counting kit-8 (CCK-8; Dojindo, Kumamoto, Japan) colorimetric assay with triplicate experiments for each set of conditions. The same amount of cells (3×10^3^ cells/well) were seeded on 96-well culture plates and cultured with/without 10 μg/ml epirubicin for 24 and 48h. At the indicated time point, the supernatant was removed, and 100 μl of DMEM medium containing 10 μl of CCK8 was added to each well for 1 h at 37°C. The absorbance at 450 nm was measured with a plate reader (Multiskan GO Microplate Spectrophotometer; Thermo Fisher Scientific, Inc., Waltham, MA, USA).

### Cell cycle assay

T24 WT and T24 Mut/T24 Mut2 were cultured with/without 10 μg/ml epirubicin for 48h, digested and collected by trypsin, fixed in 70% ethanol at −20°C overnight and then stained with 50 μg/ml propidium iodide (PI) (Sigma, St. Louis, MO, USA) and 0.1 μg/ml RNase A (Sigma, St. Louis, MO, USA). The cells were analyzed using a FACSCalibur flow cytometry system (BD Biosciences, US). Cell Quest software (BD Cell Quest Pro Software, BD Biosciences, US) was used to analyze the percentage of the cell population in each phase.

### Cellular apoptosis assay

T24 WT and T24 Mut/T24 Mut2 were cultured with/without 10 μg/ml epirubicin for 24h and 48h, and apoptosis was assessed with the Annexin V-FITC kit according to the manufacturer's instructions. The cells were washed twice with cold PBS, digested, collected, and resuspended to binding buffer. Annexin V-FITC and PI were added (BioVision, Milpitas, CA, USA), and the cells were incubated for 10 min at room temperature in the dark. Then, 200 μl binding buffer was added, and the Annexin V positive cells were analyzed using a FACSCalibur flow cytometry system (BD Biosciences, US).

### Immunohistochemistry

Urothelial bladder carcinomas were fixed in 4% formaldehyde followed by embedding in paraffin. Tissue was sectioned into 5 μm thick sections for immunohistochemistry staining. Antigen retrieval was done with heat-induced for 30min in citrate acid buffer (PH=6.0). Primary antibodies of MLL and H3K4me3 were applied overnight at 4°C, and the corresponding specific horseradish-peroxidase (HRP) labelled secondary antibodies were used for DAB coloration. The nucleus were counter stained with hematoxylin. Overall staining for MLL/H3K4me3 was measured by multiplication of staining percentage (0%–100%) and staining intensity on a numerical scale (none = 1, weak = 2, moderate = 3, strong = 4), resulting in an overall product score.

### Chromatin immunoprecipitation (ChIP)

Chromatin immunoprecipitation was performed by using the ChIP assay kit according to the manufacturer's instructions (Upstate Biotechnology). Briefly, approximately 10 million T24 WT and T24 Mut/T24 Mut2 were crosslinked with 1% formaldehyde for 10 min at 37°C, washed with PBS, and resuspended in lysis buffer. The cell lysate was sonicated on ice for a total of 2 min (in 5 s pulses), resulting in an average DNA fragment length of 500 bp. After removing cell debris by centrifugation and preclearing the lysate, immunoprecipitation was performed in ChIP dilution buffer overnight with anti-Histone H3 (tri methyl K4) antibody with agitation. Protein A agarose/Salmon Sperm DNA (Merck Millipore, Guyancourt, France) slurry was added and incubated for 2-4h at 4°C with agitation. The antibody-agarose complex was centrifuged and washed five times, and the immunoprecipitated fraction was eluted. The crosslinking was reversed by incubation at 65°C for 4h in the presence of 200 mM NaCl. The DNA was recovered by phenol/chloroform extraction and precipitated, and the abundance of specific sequence was measured by PCR using the corresponding primer sequences.

### MLL mutation by CRISPR/Cas9

The *KMT2A* (*MLL*) (Gene ID:4297) gene targeting sites in for sgRNA design was 5′-AATTCTGTCACGTTTGTGGAAGG-3′ (B112) and 5′-ACTTTGACTGTGCGTTTCCGTGG-3′ (B73) and the primers were ordered from Sangon Biotech Company (Shanghai, China). The sgRNA was inserted into two AarI site between the U6 promoter and sgRNA tails were juxtaposed right about to created two overhangs that exactly match the annealed sgRNA pair. The Cas9 sequence was consistent with previous report, and was ordered from Sangon Biotech Company (Shanghai, China). The T7 promoter was added upstream of the Cas9 coding region. All the sequences were synthesized and inserted to the T-CMV-MCS-BGPA vector to form CMV-Cas9 expression vector (Supplementary Figure 5). Genomic DNA was phenol-chloroform extracted from G418-resistant T24 cell colonies. Cell identification was carried out in PCR reactions of 50 ng genomic DNA, 10pmol of each primer (forward 5′ AATCTCCCGCAGTGTCCAAT 3′ and reverse 5′ CTCCCTCAGCCTCCCAAGTA 3′) and 0.5 unit of rTaq polymerase (Takara). PCR conditions were as follows; 5 min at 95°C ; 35 cycles of 30 sec. at 95°C, 30 sec. at 62°C, 40 sec. at 72°C ; and final extension at 72°C for 5 min. Finally, PCR products were subjected to a re-annealing process to enable heteroduplex formation: 95°C for 10 min, 95°C to 85°C ramping at −2°C/s, 85°C to 25°C at −0.25°C/s, and 16°C hold for 10 min. After re-annealing, products were treated with T7 nuclease for 1.5h, and analyzed by gel electrophoresis. Quantification was based on relative band intensities. Indel percent was determined by the formula, 100×(1−(1−(b+c)/(a+b+c))1/2), where a is the integrated intensity of the undigested PCR product, b and c are the integrated intensities of each cleavage product.

### Knockdown of GATA4 and ETS1 with shRNA

ShRNA targeted GATA4 and ETS1 and scrambled shRNAs were designed, synthesized and cloned into pSicoR-puro/GFP vector:

shCtrl: 5′-ccgg TTCTCCGAACGTGTCACGT ctcgag ACGTGACACG TTCGGAGAA tttttg-3′,

shGATA4: 5′-ccgg GACTTCTCAGAAGGCAGAG ctcgag CTCTGCCTTC TGAGAAGTC tttttg-3′;

shETS1: 5′-ccgg GCTGACCTCAATAAGGACA ctcgag TGTCCTTATT GAGGTCAGC tttttg-3′;

The resulting lentiviral vectors containing the GATA4 and ETS1 shRNA were named shGATA4 (pSicoR-puro) and shETS1 (pSicoR-GFP) lentivirus. T24 Mut cells were transfected with the shGATA4, shETS1 and both of them lentivirus to obtain cell lines stably expressing the GATA4 and ETS1 shRNA.

### Generation of xenografts

NOD/SCID mice were obtained from the Animal Center of the Chinese Academy of Medical Science (Beijing, China). For generation of xenografts, 2×10^6^ T24 WT, T24 Mut, T24 Mut shCtrl, T24 Mut shGATA4, T24 Mut shETS1 and T24 Mut Double sh were injected subcutaneously into the NOD/SCID mice (n=5). Five days later, the mice were grouped and administered intraperitoneally with DMSO or epirubicin at a dose of 2 mg/kg two times per week for 30 days. The volume of xenografts was measured every five days. Mice were sacrificed after 30 days. Animal work was permitted by the Institutional Animal Care and Use Committee (IACUC) of the Institute of Biophysics, Chinese Academy of Sciences and conducted in accordance with its recommendations and ethical regulations.

### Statistical analysis

Student's t-test was used as statistical analysis by using Microsoft Excel as described44.

### Data access

### URLs

COSMIC database, http://www.sanger.ac.uk/perl/genetics/CGP/cosmic (v52 release);

NCBI Consensus Coding Region dataset,

http://www.ncbi.nlm.nih.gov/CCDS/CcdsBrowse.cgi;

SAMtools, http://samtools.sourceforge.net/;

1000 Genomes Project, http://www.1000genomes.org/;

KEGG (Kyoto Encyclopedia of Genes and Genomes) database,

http://www.genome.jp/kegg/; MutSig, http://www.broadinstitute.org/cancer/cga/mutsig.

## SUPPLEMENTARY MATERIAL FIGURES AND TABLES








