# Supplementary material for: Novel variants in MLL confer to bladder cancer recurrence identified by whole-exome sequencing
Source: Oncotarget. 2015 Nov 25;7(3):2629–45. doi: 10.18632/oncotarget.6380 (PMC4823060; doi:10.18632/oncotarget.6380)
Supplement: Supplementary file 1 [file oncotarget-07-2629-s001.pdf]

# Novel variants in *MLL* confer to bladder cancer recurrence identified by whole-exome sequencing

**Supplementary Material**

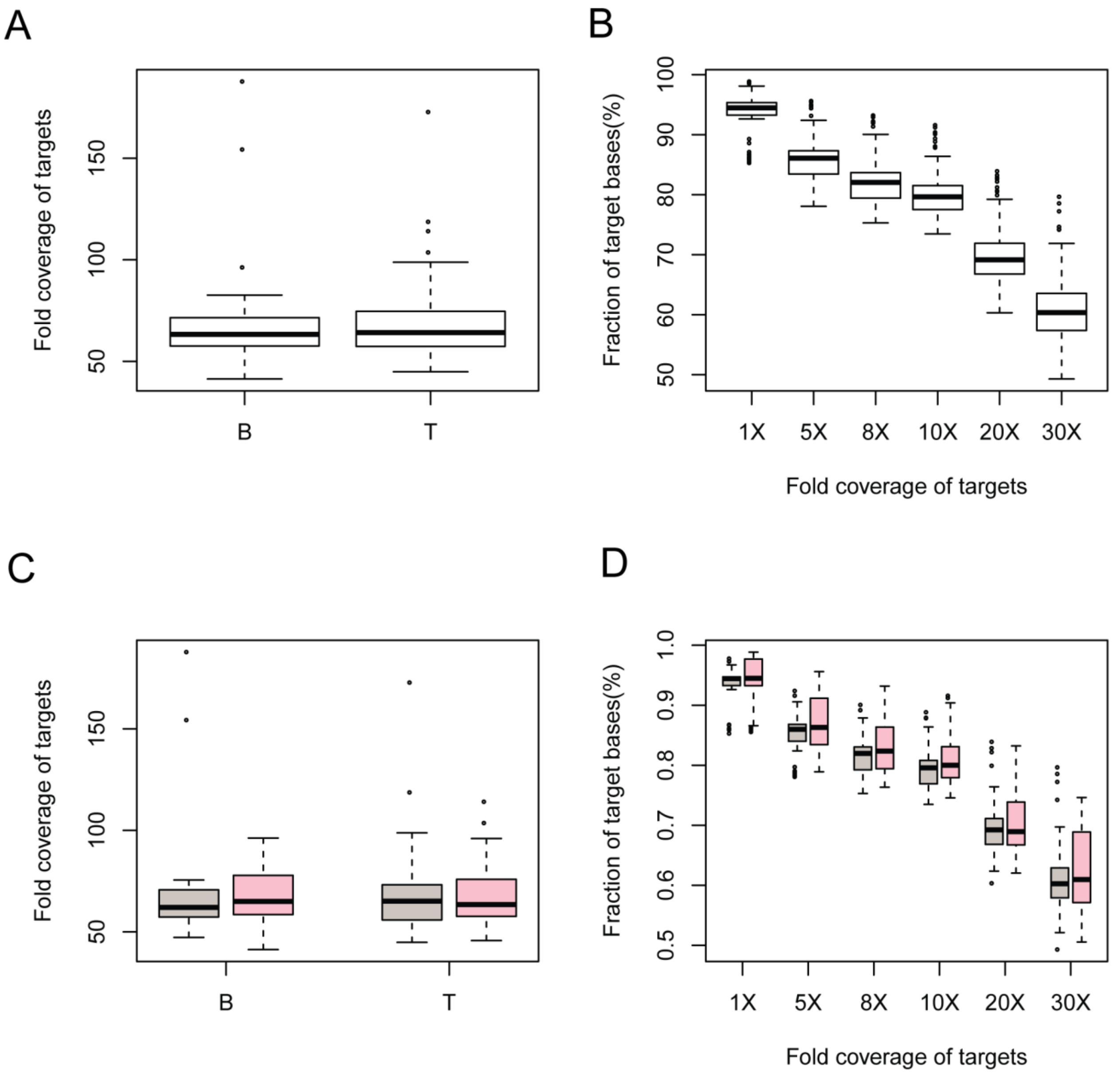


**Supplementary Figure 1. Fold coverage of target regions for the peripheral blood (B) and tumor (T) samples from 37 bladder cancer patients analyzed by WES.**
A.The box plot depicts the distribution of mean coverage of all blood and tumor samples sequenced. Lines in the two central boxes show the medians, and lines outside the two central boxes show the first and the third quartiles of the mean depths. B. The box plot depicts the distribution of fraction of targeted bases covered by at least 1×, 5×， 8×， 10× ， 20× and 30× across the 37 pairs of samples. C. The box plot depicts the distribution of mean coverage of primary (grey) and recurrent (pink) samples separately. D. The box plot depicts the distribution of fraction of targeted bases covered across primary (grey) and recurrent (pink) samples.

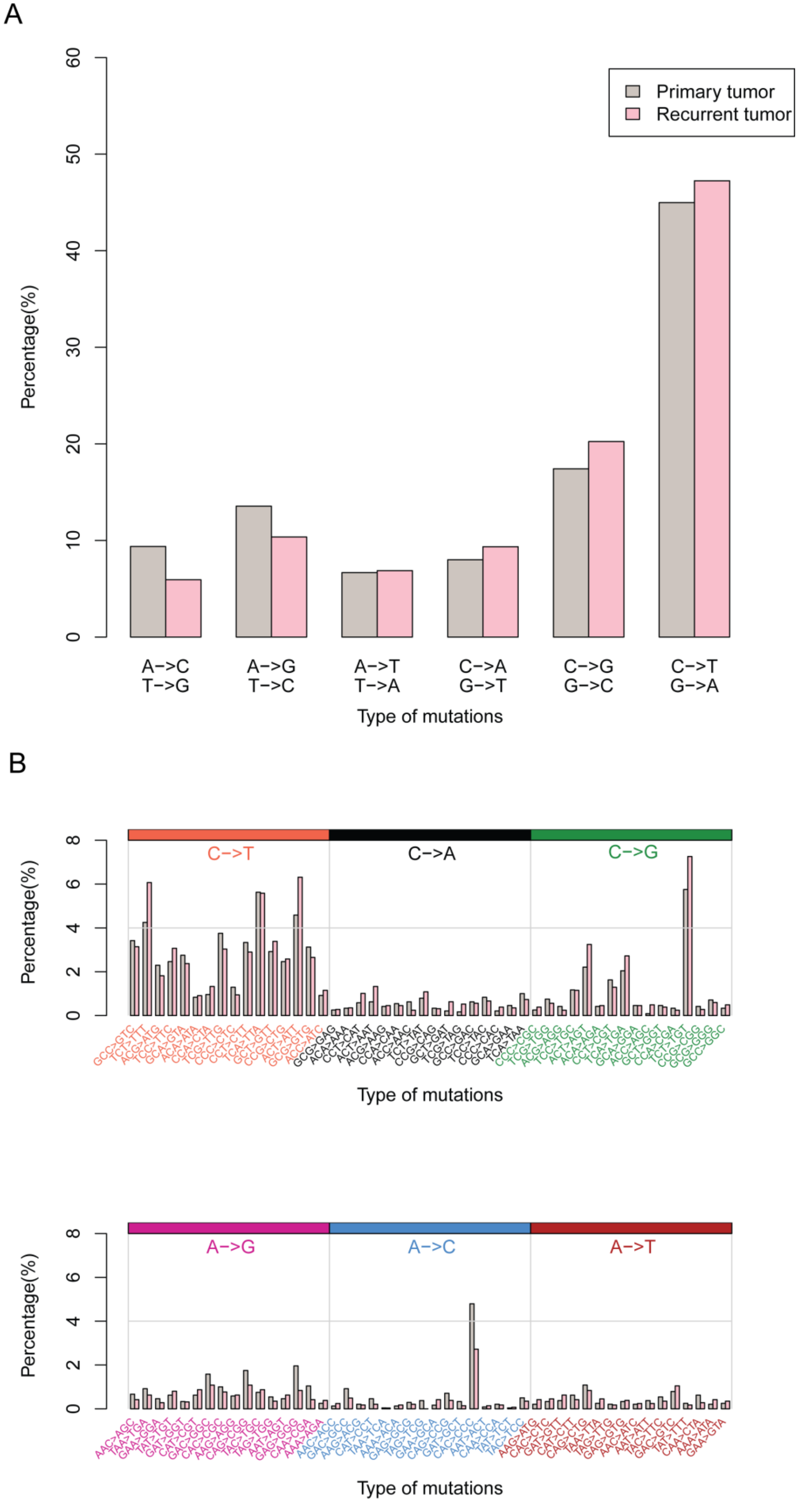


**Supplementary Figure 2. Spectrum of somatic point mutations identified in the primary and recurrent samples.**

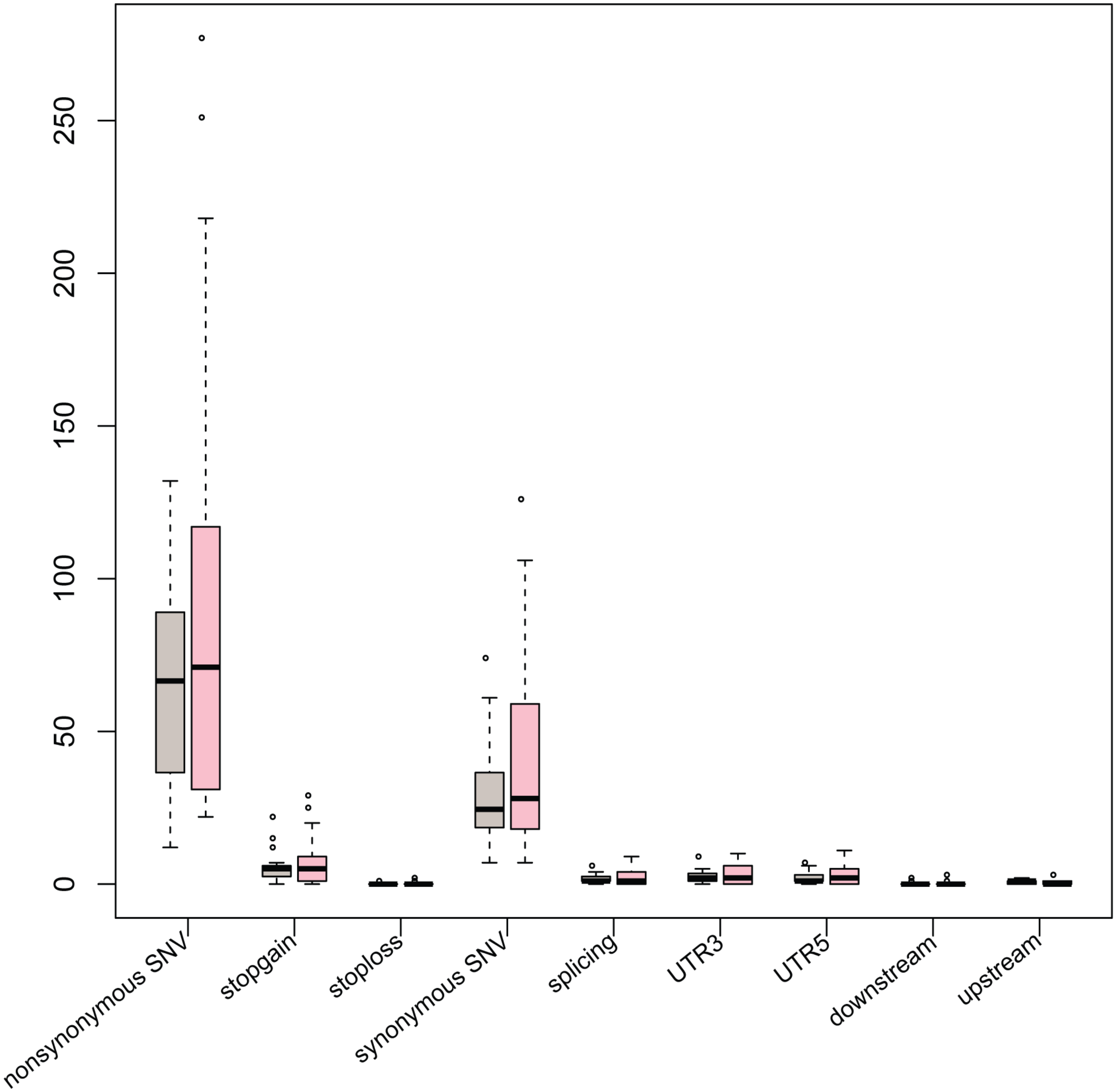


**Supplementary Figure 3. The stat of 9 types of SNV (upstream, downstream, UTR5, UTR3, splicing site, synonymous, stoploss, stopgain, and nonsynonymous ) for the primary and recurrent samples.** The box plot depicts the distribution of SNV numbers of all 20 primary samples and 17 recurrent samples parallelly. Lines in the two central boxes show the medians, and lines outside the two central boxes show the first and the third quartiles of the mean depths.

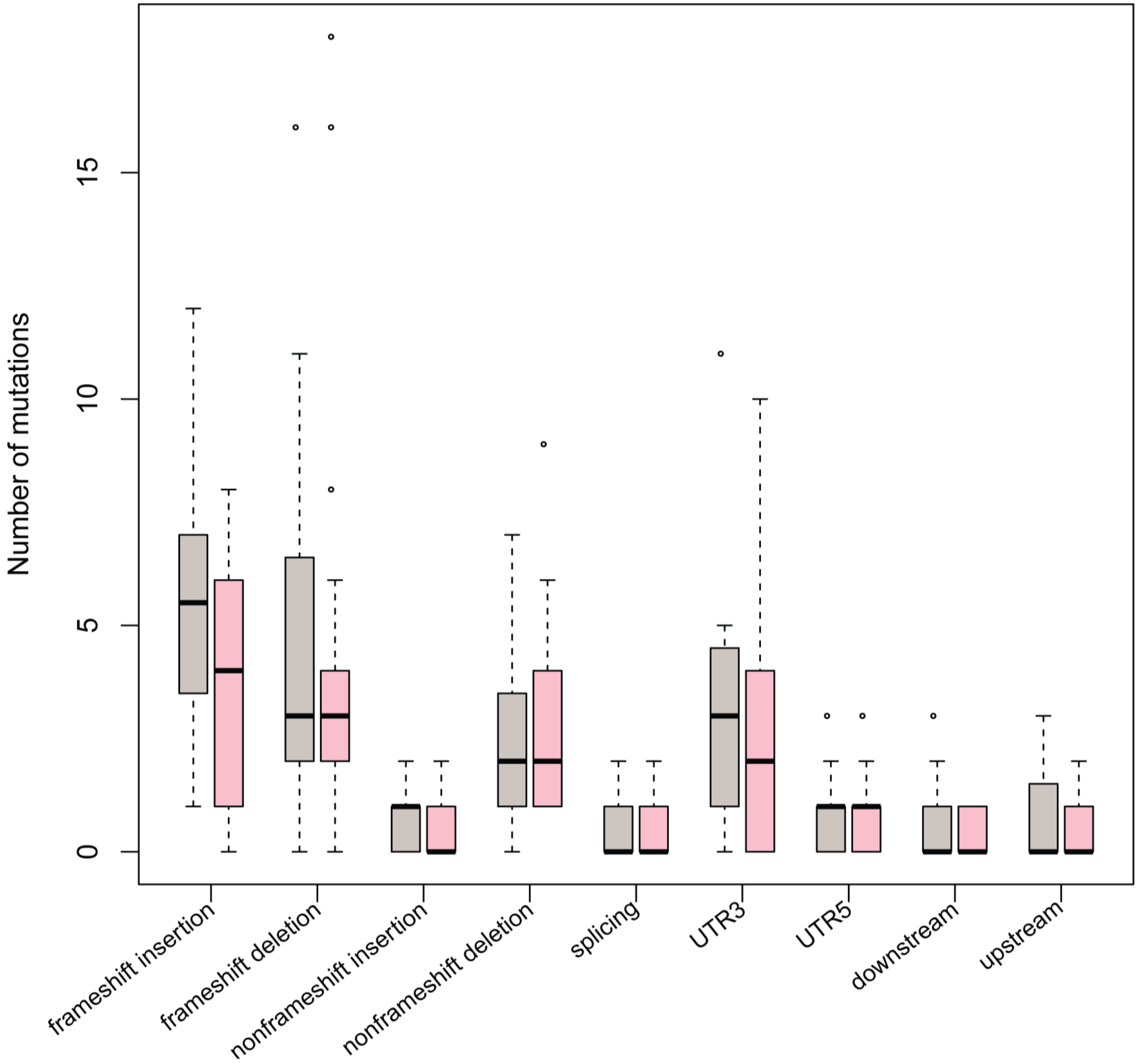


**Supplementary Figure 4. The stat of 9 types of INDEL (upstream, downstream, UTR5, UTR3, splicing site, nonframeshift deletion, nonframeshift insertion, frameshift deletion and frameshift insertion) for the primary and recurrent samples.** The box plot depicts the distribution of INDEL numbers of all 20 primary samples and 17 recurrent samples parallelly. Lines in the two central boxes show the medians, and lines outside the two central boxes show the first and the third quartiles of the mean depths.

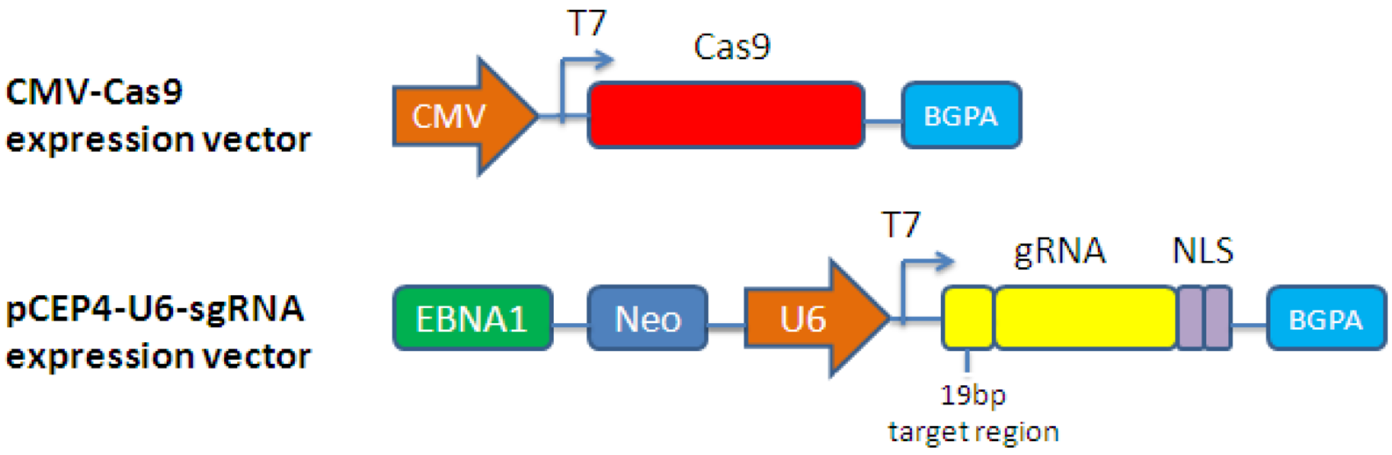


**Supplementary Figure 5. Schematic representation of the sgRNA and Cas9 expression vectors.** T24 cell were seeded into 6-well plates before transfection, the CMV-Cas9 expression vector and pCEP4-U6-sgRNA expression vector were co-transfected using Lipofectamine 2000 following the recommended protocols when the cell reach 80%-90% confluency. The total of 2ug Cas9+sgRNA vector was used at 1:1 quality ratio for each well. 24 h after transfection, one well of cells were seeded into eight 10-cm cell culture dishes. The cell was added 500ug/ml G418 at the density of 30%-40% and screened for about one week until the G418-resistant cell clones form -ed. The clones were trypsinized and transferred into 48-well for PCR screening.

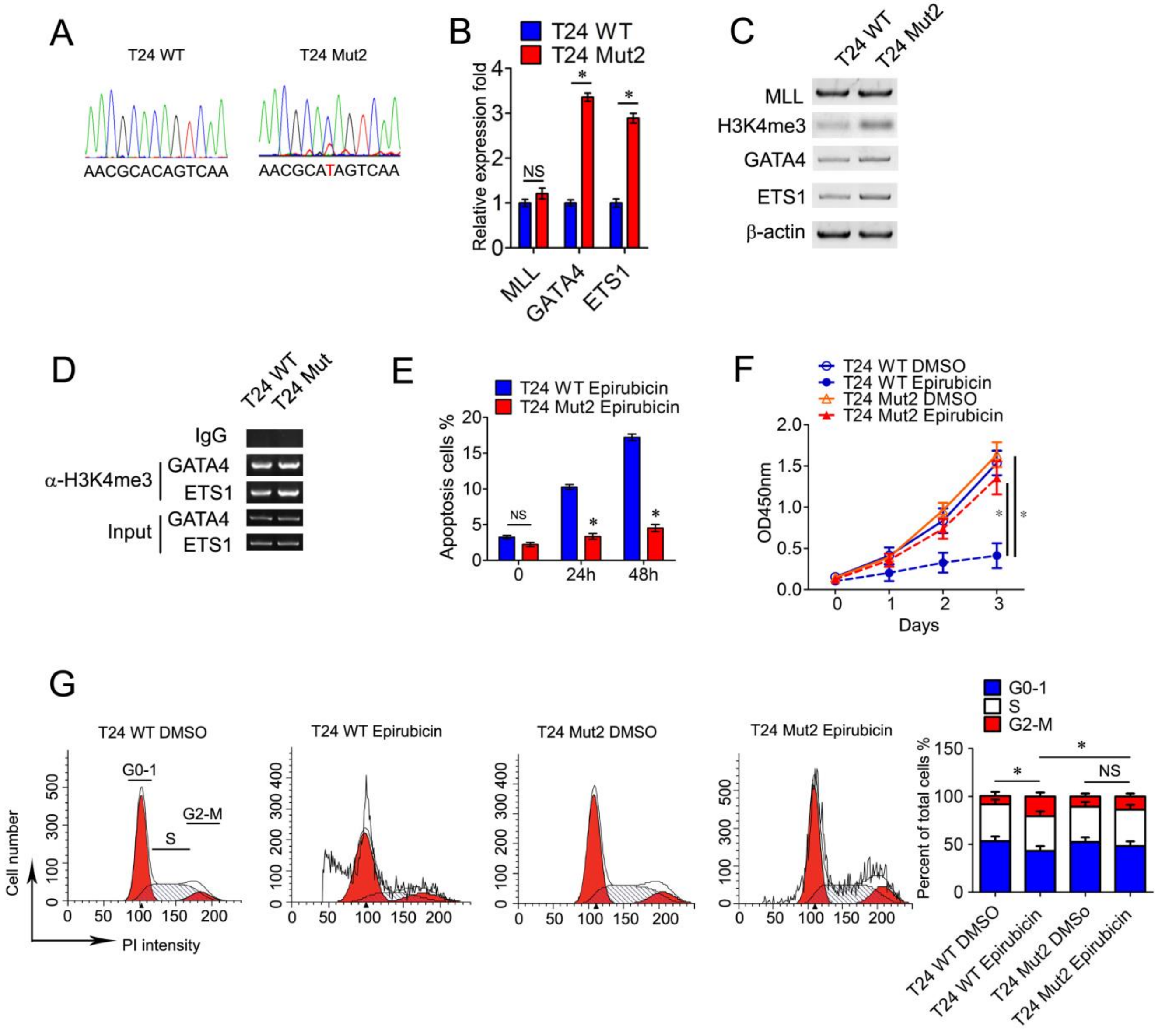


Supplementary Figure 6. *MLL* mutation g.chr11:118374172C>T enhanced the drug-resistance of T24 cells to epirubicin.

A. Sanger sequencing of *MLL* PCR product in T24 WT and T24 Mut2. B. Real-time PCR analysis of MLL, GATA4, and ETS1 mRNA levels in T24 WT and T24 Mut2. Data is displayed as mean±SD. C. Western blot analysis of MLL, GATA4 and ETS1 in T24 WT and T24 Mut2. β-actin was used as loading control.

D. CHIP assay was performed using H3K4me3 antibody and IgG antibody in T24 WT and T24 Mut2.

E. Epirubicin was applied to induce apoptosis in T24 WT and T24 Mut2 cells and the cells were collected and analyzed at indicated point. Data is expressed as mean±SD. F The propagation curves of T24 WT and T24 Mut were measured by CCK8 with/without the treatment of epirubicin. Data is displayed as mean±SD. G. The cell cycle of T24 WT and T24 Mut were measured by PI staining with/without the treatment of epirubicin. Data is showed as mean ± SD. ∗P<0.05.
