# Supplementary material for: Novel variants in MLL confer to bladder cancer recurrence identified by whole-exome sequencing
Source: Oncotarget. 2015 Nov 25;7(3):2629–45. doi: 10.18632/oncotarget.6380 (PMC4823060; doi:10.18632/oncotarget.6380)
Supplement: Supplementary file 2 [file oncotarget-07-2629-s002.pdf]

Supplementary Table 1. Clinical characteristics of 37 bladder cancer patients.

Patient ID	Patient age (years)	Sex	Stage (TNM classifcation*)	Grade	Primary/Recurrent
B105	62	M	T2N0M0	high	Recurrent
B65	56	M	T3aN0M0	high	Recurrent
B80-1	58	M	T2N0M0	high	Recurrent
B98	63	M	T3N1M0	low	Recurrent
B109	46	M	TaN0M0	low	Recurrent
B11	65	M	T4N0M0	high	Recurrent
B25	65	M	T4aN0M0	high	Recurrent
B71	65	M	T2N0M0	low	Recurrent
B96	69	M	T3N0M0	high	Recurrent
B73	44	M	TaN0M0	low	Recurrent
B80-5	73	M	TaN0M0	low	Recurrent
B112	75	M	TaN0M0	low	Recurrent
W67	74	M	T1N0M0	low	Recurrent
W68	71	M	T1N0M0	high	Recurrent
W70	54	F	T1N0M0	high	Recurrent
W72	80	M	T1N0M0	high	Recurrent
chencian	75	M	T3bN0M0	high	Recurrent
B57	72	F	T2aN0M0	low	Primary
B59-3	65	M	T2N0M0	low	Primary
B68	85	M	T2N0M0	low	Primary
B64	66	M	TaN0M0	low	Primary
B66-0	50	F	TaN0M0	high	Primary
B78	78	M	TaN0M0	low	Primary
B89-10	65	M	TaN0M0	high	Primary
B21	61	M	T2N0M0	low	Primary
B70	65	M	T2N0M0	high	Primary
B80-0	76	M	TaN0M0	low	Primary
B80-13	63	M	TaN0M0	high	Primary
B59-0	50	M	T2N0M0	low	Primary
B59-1	43	M	T1N0M0	low	Primary
B45	82	M	TaN0M0	high	Primary
B56	41	M	TaN0M0	low	Primary
B62-0	58	M	TaN0M0	high	Primary
B80-11	28	M	TaN0M0	low	Primary
B89-5	50	M	TaN0M0	high	Primary
B99	37	F	T3N0M0	low	Primary
B77	75	M	T1N0M0	high	Primary

* The TNM cancer staging system was designed to gauge the extent of cancer in a patient's body. T describes the size of the tumor and whether it has invaded nearby tissue, N describes regional lymph nodes that are involved, and M describes distant metastasis (spread of cancer from one body part to another).

Supplementary Table 2. Summary statistics of exome sequencing data obtained from the 20 primary bladder cancer patients.

Patient ID	Sample	Total reads	NO. of uniquely mapping reads	% of uniquely mapping reads	NO.of reads overlapping targets	% of reads overlapping targets	NO.of non-duplicated reads	Mean fold coverage	% of targets covered by at least 1X	% of targets covered by at least 10X	% of targets covered by at least 30X
B21	peripheral blood	48690545	42871015	88.047926	37439786	76.89	37385383	64.46	94.75	80.62	61.24
	tumor	59487105	52526094	88.298286	43220295	72.65	43177925	71.82	94.65	80.9	62.72
B45	peripheral blood	58777387	52025900	88.513462	44717528	76.08	44677411	75.54	94.6	81.51	64.38
	tumor	45046526	39831899	88.423909	34915185	77.51	34884810	59.31	94.06	79.08	58.76
B56	peripheral blood	46989685	41661154	88.660211	34748836	73.95	34737947	58.63	94.29	79.49	59.16
	tumor	46650304	41357090	88.65342	36132719	77.45	36115906	61.59	94.09	79.27	59.02
B57	peripheral blood	64667175	57809318	89.39515	44017356	68.07	44005616	72.25	94.69	80.33	62.31
	tumor	72098051	64335091	89.232774	51937822	72.04	51917135	87.01	95.41	82.65	66.86
B59-0	peripheral blood	167080953	149547513	89.506021	113349553	67.84	113271393	187.79	97.77	88.83	79.65
	tumor	49125439	43472530	88.492909	37187412	75.70	37164910	63.52	94.62	80.12	60.44
B59-1	peripheral blood	137649299	123185036	89.491946	93517884	67.94	93456579	154.29	97.35	87.83	77.22
	tumor	44183802	39259877	88.855814	32869941	74.39	32856148	54.89	93.91	78.39	57.21
B59-3	peripheral blood	49031727	43202843	88.112016	37548743	76.58	37527080	64.04	94.44	80.16	60.84
	tumor	76507731	67485052	88.20684	58015306	75.83	57969450	98.79	95.83	84.03	69.72
B62-0	peripheral blood	44384037	39027182	87.930672	33997333	76.60	33975303	57.54	93.97	78.93	58.24
	tumor	40626585	35735130	87.959965	32033281	78.85	32012141	54.91	93.71	78.32	56.65
B64	peripheral blood	52803660	47168486	89.328062	35414529	67.07	35402991	58.72	94.49	78.43	58.04
	tumor	59111245	52792786	89.310902	41410464	70.06	41393102	69.58	94.83	80.32	61.88
B66-0	peripheral blood	57736322	51378470	88.988124	42532843	73.67	42511993	71.36	94.84	81.1	63.48
	tumor	43046702	38361170	89.115236	32254784	74.93	32240370	54.2	93.51	77.54	56.29
B68	peripheral blood	49754669	44002011	88.437953	37266518	74.90	37250511	62.79	94.44	80.04	60.71
	tumor	50945337	45315819	88.949886	39067520	76.69	39047111	66.07	94.13	79.65	60.23
B70	peripheral blood	42232983	37389630	88.531824	32049110	75.89	32036603	54.19	94.38	79.04	57.38
	tumor	99480442	88649068	89.112057	71259056	71.63	71224108	118.68	96.73	86.38	74.23
B77	peripheral blood	37458705	33247122	88.756731	28287865	75.52	28278892	47.3	93.03	75.17	52.11
	tumor	34070890	30246295	88.774596	26534101	77.88	26524182	44.84	92.62	73.88	49.29

B78	peripheral blood	47592512	42451404	89.197654	34445131	72.38	34435251	57.88	94.72	79.1	58.49
	tumor	61619658	55300355	89.744664	44514899	72.24	44497727	74.6	95.35	81.35	63.56
B80-0	peripheral blood	47754749	42286920	88.550188	36089699	75.57	36072069	61.35	94.66	80.05	60.2
	tumor	52649903	46224412	87.795816	40178117	76.31	40151099	68.86	94.46	79.89	60.27
B80-11	peripheral blood	54957784	49367950	89.828858	41333136	75.21	41314816	70.04	86.75	76.29	62.34
	tumor	43149143	38741707	89.785577	33111771	76.74	33098262	56.4	85.88	74.93	57.76
B80-13	peripheral blood	46716227	42031132	89.971161	34086150	72.96	34074349	57.14	86.13	74.65	58.05
	tumor	43464970	39111213	89.983297	32681825	75.19	32668975	55.36	86.01	74.67	57.45
B89-10	peripheral blood	42737041	38427418	89.915954	31909494	74.66	31888474	53.78	85.28	73.8	56.22
	tumor	52939875	47612645	89.937207	40913711	77.28	40873397	70.27	86.66	76.28	61.94
B89-5	peripheral blood	45711335	41238505	90.215053	32903604	71.98	32892719	55.25	85.87	73.48	56.44
	tumor	51071723	45884850	89.843944	37998968	74.40	37981924	64.16	86.01	74.58	59.38
B99	peripheral blood	52741646	46929846	88.980625	40334532	76.48	40290346	68.68	94.51	80.72	63.06
	tumor	140501992	125737468	89.491591	102783026	73.15	102642730	172.77	97.28	87.91	78.55

Supplementary Table 3. Summary statistics of exome sequencing data obtained from the 17 recurrent bladder cancer patients.

Patient ID	Sample	Total reads	NO. of uniquely mapping reads	% of uniquely mapping reads	NO.of reads overlapping targets	% of reads overlapping targets	NO.of non-duplicated reads	Mean fold coverage	% of targets covered by at least 1X	% of targets covered by at least 10X	% of targets covered by at least 30X
B105	peripheral blood	37500548	33275151	88.73	28613977	76.30	28602095	49.14	93.6	76.56	53.15
	tumor	34163495	30270455	88.60	26458671	77.45	26445635	45.73	93.26	75.47	50.55
B109	peripheral blood	47181321	41732855	88.45	35924422	76.14	35906525	61.9	94.5	79.64	59.6
	tumor	60487207	53450846	88.37	45124465	74.60	45105293	75.88	95.35	82.37	65.57
B112	peripheral blood	52343024	46403424	88.65	37752261	72.12	37736477	63.24	94.97	80.56	61.34
	tumor	54983654	48674405	88.53	41169246	74.88	41148521	69.54	95.11	81.52	63.48
B11	peripheral blood	46665643	41349540	88.61	34647057	74.25	34630336	58.53	94.26	78.67	58.01
	tumor	46430687	41021602	88.35	35181858	75.77	35158843	59.25	93.79	77.93	57.27
B25	peripheral blood	58633085	51581352	87.97	42860658	73.10	42809834	71.5	94.72	80.93	62.78
	tumor	51593229	45328260	87.86	39423086	76.41	39358114	66.83	94.4	80.37	61.41
B65	peripheral blood	47674102	42537638	89.23	33139874	69.51	33129044	54.58	94.47	78.41	57.12
	tumor	50599805	45186876	89.30	35760281	70.67	35747928	59.1	94.47	78.53	57.37
B71	peripheral blood	53324333	47541007	89.15	38993264	73.12	38981421	64.98	94.46	79.44	60.29
	tumor	93956748	83795096	89.18	68324649	72.72	68290593	114.07	96.09	84.82	71.42
B73	peripheral blood	56788999	50365739	88.69	42012047	73.98	41991665	70.59	94.93	81.13	63.04
	tumor	42550519	37685591	88.57	32655630	76.75	32640529	55.17	93.83	78.17	56.91
B80-1	peripheral blood	51366325	46248876	90.04	37019911	72.07	37001671	62.36	87.1	75.73	60.56
	tumor	77700562	69656632	89.65	59387746	76.43	59291998	103.6	89.27	78.57	68.89
B80-5	peripheral blood	40539502	36231560	89.37	30950258	76.35	30935602	53.04	86.11	74.57	55.72
	tumor	43622631	38856682	89.07	33715224	77.29	33694960	57.63	85.85	74.63	56.77
B96	peripheral blood	73994541	66235741	89.51	56228608	75.99	56172641	96.21	88.58	78.34	68.32
	tumor	46850444	41905812	89.45	36847966	78.65	36813122	63.49	86.32	75.48	59
B98	peripheral blood	51499739	46345229	89.99	39159321	76.04	39127581	66.88	86.6	76.16	61.55
	tumor	42977339	38698554	90.04	33765760	78.57	33735896	58.03	85.54	75.04	58.09
W100	peripheral blood	32722796	28963127	88.51	25161259	76.89	25130182	41.29	97.16	81.8	52.11
	tumor	54090178	48345627	89.38	43008038	79.51	42940668	70.93	98.1	88.05	68.88

W67	peripheral blood	60081553	52257107	86.98	46097935	76.73	45950668	81.06	98.69	90.39	71.88
	tumor	65721409	57401333	87.34	50489059	76.82	50323003	88.78	98.85	91.58	74.63
W68	peripheral blood	60022140	51991637	86.62	45524395	75.85	45385936	80.15	98.76	90.23	70.86
	tumor	39805437	34323251	86.23	31400774	78.89	31270248	55.85	97.87	83.09	55.03
W70	peripheral blood	61668908	53966817	87.51	46849544	75.97	46714992	82.59	98.51	90.09	71.29
	tumor	70570574	61906815	87.72	54509540	77.24	54347830	95.98	98.73	91.21	74.16
W72	peripheral blood	58332769	51138344	87.67	44071368	75.55	43935643	77.84	98.57	89.34	69.33
	tumor	42119100	36703429	87.14	32082284	76.17	31825926	57.34	97.7	83.1	55.35

Supplementary Table 4. The detailed information of somatic SNV and INDEL validation.

Tumor ID	Function	Gene Symbol	Exon Function	Nucleotide (genomic)#	Nucleotide (cDNA)$	Amino acid (protein)
B105	exonic	KDM6A	nonsynonymous SNV	g.chrX:44918524A>G	c.A1007G	p.D336G
B96	exonic	KDM6A	stopgain SNV	g.chrX:44922802C>T	c.C1663T	p.Q555X
B25	exonic	KDM6A	frameshift deletion	g.chrX:44920644TTGA>-	c.1405_1408del	p.469_470del
B25	exonic	KDM6A	frameshift deletion	g.chrX:44920650CCAC>-	c.1411_1414del	p.471_472del
B25	exonic	KDM6A	frameshift deletion	g.chrX:44920656AA>-	c.1417_1418del	p.473_473del
B57	exonic	KDM6A	stopgain SNV	g.chrX:44938411A>T	c.A2959T	p.K987X
B57	exonic	KDM6A	stopgain SNV	g.chrX:44942817C>T	c.C3397T	p.Q1133X
B89-5	exonic	KDM6A	stopgain SNV	g.chrX:44833954C>G	c.C378G	p.Y126X
B45	exonic	KDM6A	frameshift deletion	g.chrX:44928977GGGGCTGC>-	c.2077_2084del	p.693_695del
B112	exonic	TP53	nonsynonymous SNV	g.chr17:7578458G>A	c.C76T	p.R26C
B80-1	exonic	TP53	nonsynonymous SNV	g.chr17:7572980T>G	c.A733C	p.T245P
B96	exonic	TP53	stopgain SNV	g.chr17:7577022G>A	c.C520T	p.R174X
B59-3	exonic	TP53	stopgain SNV	g.chr17:7579533G>A	c.C154T	p.Q52X
B77	exonic	TP53	nonsynonymous SNV	g.chr17:7577085C>T	c.G457A	p.E153K
B80-0	exonic	TP53	nonsynonymous SNV	g.chr17:7578534C>G	c.G396C	p.K132N
B105	exonic	CREBBP	stopgain SNV	g.chr16:3807902G>A	c.C3403T	p.R1135X
B109	exonic	CREBBP	nonsynonymous SNV	g.chr16:3788618G>C	c.C4222G	p.R1408G
B98	exonic	CREBBP	synonymous SNV	g.chr16:3778535C>T	c.G6399A	p.L2133L
B62-0	exonic	CREBBP	stopgain SNV	g.chr16:3808901G>T	c.C3209A	p.S1070X
B80-13	exonic	CREBBP	stopgain SNV	g.chr16:3820936G>A	c.C2401T	p.Q801X
B59-0	exonic	CREBBP	frameshift deletion	g.chr16:3820766G>-	c.2571delC	p.S857fs

B78	exonic	EP300	stopgain SNV	g.chr22:41545855C>T	c.C2470T	p.Q824X
B80-13	exonic	EP300	nonsynonymous SNV	g.chr22:41569671G>C	c.G4662C	p.K1554N
B89-10	exonic	EP300	nonframeshift deletion	g.chr22:41566500GCCCAAGCGACT>-	c.4377_4388del	p.1459_1463del
B98	exonic	HRAS	nonsynonymous SNV	g.chr11:534289C>T	c.G34A	p.G12S
B59-1	exonic	HRAS	nonsynonymous SNV	g.chr11:534289C>T	c.G34A	p.G12S
B62-0	exonic	HRAS	nonsynonymous SNV	g.chr11:533874T>C	c.A182G	p.Q61R
B68	exonic	HRAS	nonsynonymous SNV	g.chr11:533874T>A	c.A182T	p.Q61L
B70	exonic	HRAS	nonsynonymous SNV	g.chr11:534286C>G	c.G37C	p.G13R
B80-11	exonic	HRAS	nonsynonymous SNV	g.chr11:533874T>C	c.A182G	p.Q61R
B99	exonic	HRAS	nonsynonymous SNV	g.chr11:534288C>T	c.G35A	p.G12D
W100	exonic	ERBB3	synonymous SNV	g.chr12:56487222A>T	c.A1368T	p.I456I
B80-0	exonic	ERBB3	synonymous SNV	g.chr12:56490951G>C	c.G2397C	p.V799V
B65	exonic	CHD6	stopgain SNV	g.chr20:40116442T>A	c.A1864T	p.K622X
B65	exonic	LRP2	nonsynonymous SNV	g.chr2:170099937C>G	c.G3526C	p.D1176H
B68	exonic	LRP2	nonsynonymous SNV	g.chr2:170062612T>C	c.A7477G	p.M2493V
B112	exonic	FGFR3	nonsynonymous SNV	g.chr4:1803564C>T	c.C742T	p.R248C
B98	exonic	FGFR3	nonsynonymous SNV	g.chr4:1803564C>T	c.C742T	p.R248C
W68	exonic	FGFR3	nonsynonymous SNV	g.chr4:1806099A>G	c.A1118G	p.Y373C
B56	exonic	FGFR3	nonsynonymous SNV	g.chr4:1803669C>T	c.C847T	p.P283S
B56	exonic	FGFR3	nonsynonymous SNV	g.chr4:1807889A>G	c.A1612G	p.K538E
B64	exonic	FGFR3	nonsynonymous SNV	g.chr4:1806099A>G	c.A1118G	p.Y373C
B66-0	exonic	LAMA4	nonsynonymous SNV	g.chr6:112493865C>T	c.G1499A	p.R500K
B65	exonic	ANK2	nonsynonymous SNV	g.chr4:114278181G>A	c.G8407A	p.E2803K
B70	exonic	ANK2	nonsynonymous SNV	g.chr4:114274838G>C	c.G5064C	p.Q1688H
B96	exonic	ESPL1	nonsynonymous SNV	g.chr12:53670572G>C	c.G1869C	p.W623C
B96	exonic	NCOR1	nonsynonymous SNV	g.chr17:15973643G>A	c.C4397T	p.S1466L

B96	exonic	NCOR1	stopgain SNV	g.chr17:16012202G>A	c.C1753T	p.R585X
B45	exonic;splicing	NCOR1	nonsynonymous SNV	g.chr17:15995176C>A	c.G2738T	p.G913V
B45	exonic	KRAS	nonsynonymous SNV	g.chr12:25398284C>A	c.G35T	p.G12V
B71	exonic	MLL3	synonymous SNV	g.chr7:151860015C>A	c.G10647T	p.V3549V
B70	exonic	MLL3	stopgain SNV	g.chr7:151873504G>A	c.C9034T	p.Q3012X
B78	exonic	MLL3	nonsynonymous SNV	g.chr7:151919751A>G	c.T3340C	p.C1114R
B25	exonic	PIK3CA	nonsynonymous SNV	g.chr3:178936082G>A	c.G1624A	p.E542K
B65	exonic	PIK3CA	nonsynonymous SNV	g.chr3:178936091G>A	c.G1633A	p.E545K
B98	exonic	PIK3CA	nonsynonymous SNV	g.chr3:178936082G>A	c.G1624A	p.E542K
B98	exonic	PIK3CA	nonsynonymous SNV	g.chr3:178936091G>C	c.G1633C	p.E545Q
B45	exonic	PIK3CA	nonsynonymous SNV	g.chr3:178916876G>A	c.G263A	p.R88Q
B64	exonic	PIK3CA	nonsynonymous SNV	g.chr3:178936091G>A	c.G1633A	p.E545K
B70	exonic	PIK3CA	nonsynonymous SNV	g.chr3:178936091G>C	c.G1633C	p.E545Q
B78	exonic	PIK3CA	nonsynonymous SNV	g.chr3:178952085A>G	c.A3140G	p.H1047R
B57	exonic	PIK3CA	nonframeshift deletion	g.chr3:178916938GAA>-	c.325_327del	p.109_109del
B112	exonic	TPR	nonsynonymous SNV	g.chr1:186316571T>G	c.A2796C	p.E932D
W72	exonic	TPR	nonsynonymous SNV	g.chr1:186294980C>T	c.G6028A	p.D2010N
B89-10	exonic	FBXW7	synonymous SNV	g.chr4:153249467T>A	c.A957T	p.G319G
B105	exonic	STAG2	stopgain SNV	g.chrX:123220428C>T	c.C3085T	p.Q1029X
B109	exonic	STAG2	nonsynonymous SNV	g.chrX:123210212T>C	c.T2564C	p.I855T
B57	exonic	STAG2	stopgain SNV	g.chrX:123171394G>A	c.G306A	p.W102X
B105	exonic	EP400	nonsynonymous SNV	g.chr12:132445775G>A	c.G611A	p.G204E
B109	exonic	EP400	nonsynonymous SNV	g.chr12:132562211G>A	c.G9365A	p.C3122Y
B11	splicing	EP400	-	g.chr12:132475951G>-	-	-
B25	exonic	EP400	nonsynonymous SNV	g.chr12:132527953G>T	c.G6312T	p.M2104I
B66-0	exonic	EP400	synonymous SNV	g.chr12:132445896C>T	c.C732T	p.A244A

B112	exonic	MLL	nonsynonymous SNV	g.chr11:118359433C>G	c.C4437G	p.C1479W
B71	exonic	MLL	nonsynonymous SNV	g.chr11:118375263C>G	c.C8656G	p.L2886V
B73	exonic	MLL	nonsynonymous SNV	g.chr11:118374172C>T	c.C7565T	p.T2522I
W100	exonic	MLL	nonsynonymous SNV	g.chr11:118348796G>A	c.G3449A	p.R1150H
B96	exonic	PRDM2	frameshift insertion	g.chr1:14107960->TTGAAAGC	c.3067_3068insTTGAAAGC	p.F1023fs
B98	exonic	PRDM2	nonsynonymous SNV	g.chr1:14106927G>C	c.G2034C	p.K678N
W68	exonic	PRDM2	nonsynonymous SNV	g.chr1:14106020A>T	c.A1127T	p.N376I
W100	exonic	PRDM2	stopgain SNV	g.chr1:14104972C>T	c.C79T	p.Q27X
B65	exonic	ANK3	nonsynonymous SNV	g.chr10:61833553C>G	c.G7086C	p.Q2362H
B71	exonic	ANK3	nonsynonymous SNV	g.chr10:61834952G>C	c.C5687G	p.S1896C
B96	exonic	ANK3	nonsynonymous SNV	g.chr10:61967820T>G	c.A1117C	p.K373Q
B56	exonic	ANK3	synonymous SNV	g.chr10:61829521T>G	c.A11118C	p.A3706A
B112	exonic	CHD5	nonsynonymous SNV	g.chr1:6170581C>A	c.G5255T	p.G1752V
B112	exonic	CHD5	nonsynonymous SNV	g.chr1:6170582C>A	c.G5254T	p.G1752C
B109	exonic	CHD5	nonsynonymous SNV	g.chr1:6171937T>C	c.A5147G	p.H1716R
B109	exonic	CHD5	nonsynonymous SNV	g.chr1:6202568G>C	c.C2141G	p.S714C
B25	exonic	CHD5	nonsynonymous SNV	g.chr1:6188977C>A	c.G3540T	p.K1180N
B65	exonic	CHD5	synonymous SNV	g.chr1:6215673G>A	c.C492T	p.F164F
B71	exonic	CHD5	synonymous SNV	g.chr1:6190315C>T	c.G3336A	p.A1112A

Supplementary Table 5. Differential analysis of mutated genes in recurrent and primary bladder cancer patients.

Gene	Recurrent sample number	Primary sample number	p-value
MLL	4	0	0.04
EP400	4	0	0.04
PRDM2	4	0	0.04
ANK3	3	0	0.09
CHD5	3	0	0.09
MLL3	0	2	0.49
EP300	0	3	0.23
FGFR3	3	2	0.64
HRAS	1	6	0.10
TP53	4	3	0.68
RB1	2	1	0.58

Supplementary Table 6. Clinical characteristics of additional 80 bladder cancer patients.

Patient ID	Patient age (years)	Sex	Stage (TNM classifcation*)	Grade	Primary/Recurrent	*MLL* matation
SR1	58	M	TaN0M0	low	Recurrent	×
SR2	63	M	T1N0M0	low	Recurrent	×
SR3	54	M	TaN0M0	low	Recurrent	×
SR4	43	F	TaN0M0	low	Recurrent	×
SR5	59	M	TaN0M0	low	Recurrent	g.chr11:118375263C>G
SR6	46	M	T1N0M0	low	Recurrent	×
SR7	49	M	TaN0M0	low	Recurrent	×
SR8	61	M	TaN0M0	low	Recurrent	×
SR9	73	M	TaN0M0	low	Recurrent	×
SR10	53	M	T1N0M0	low	Recurrent	×
SR11	46	M	TaN0M0	low	Recurrent	×
SR12	59	M	TaN0M0	low	Recurrent	×
SR13	47	F	TaN0M0	low	Recurrent	×
SR14	59	M	TaN0M0	low	Recurrent	g.chr11:118359433C>G
SR15	65	M	TaN0M0	low	Recurrent	×
SR16	58	M	TaN0M0	low	Recurrent	×
SR17	57	M	TaN0M0	low	Recurrent	g.chr11:118374172C>T
SR18	67	F	TaN0M0	low	Recurrent	×
SR19	56	M	TaN0M0	low	Recurrent	×
SR20	58	M	T1N0M0	low	Recurrent	×
SR21	56	M	TaN0M0	low	Recurrent	×
SR22	75	F	TaN0M0	low	Recurrent	×
SR23	58	M	TaN0M0	low	Recurrent	g.chr11:118348796G>A
SR24	56	M	TaN0M0	low	Recurrent	×
SR25	65	M	TaN0M0	low	Recurrent	g.chr11:118374172C>T
SR26	56	M	T1N0M0	low	Recurrent	×
SR27	67	M	TaN0M0	low	Recurrent	×
SR28	59	M	TaN0M0	low	Recurrent	×
SR29	52	M	TaN0M0	low	Recurrent	g.chr11:118359433C>G
SR30	53	F	TaN0M0	low	Recurrent	×
SR31	73	M	TaN0M0	low	Recurrent	×
SR32	45	M	T1N0M0	low	Recurrent	×
SR33	76	M	TaN0M0	low	Recurrent	g.chr11:118359433C>G
SR34	53	M	TaN0M0	low	Recurrent	×
SR35	58	M	TaN0M0	low	Recurrent	×
SR36	39	F	TaN0M0	low	Recurrent	×
SR37	57	M	T1N0M0	low	Recurrent	×
SR38	72	M	TaN0M0	low	Recurrent	×
SR39	69	M	TaN0M0	low	Recurrent	×
SR40	71	M	T1N0M0	low	Recurrent	×

SP1	45	M	TaN0M0	low	Primary	×
SP2	68	M	TaN0M0	low	Primary	×
SP3	73	M	T1N0M0	low	Primary	×
SP4	72	M	TaN0M0	low	Primary	×
SP5	63	M	TaN0M0	low	Primary	×
SP6	53	M	T1N0M0	low	Primary	×
SP7	64	F	TaN0M0	low	Primary	×
SP8	45	M	TaN0M0	low	Primary	×
SP9	73	M	TaN0M0	low	Primary	×
SP10	75	M	T1N0M0	low	Primary	×
SP11	46	M	TaN0M0	low	Primary	×
SP12	59	M	TaN0M0	low	Primary	×
SP13	80	M	T1N0M0	low	Primary	×
SP14	45	F	TaN0M0	low	Primary	×
SP15	71	M	TaN0M0	low	Primary	×
SP16	58	M	TaN0M0	low	Primary	×
SP17	66	M	TaN0M0	low	Primary	×
SP18	53	M	T1N0M0	low	Primary	×
SP19	75	M	TaN0M0	low	Primary	×
SP20	69	M	TaN0M0	low	Primary	×
SP21	58	M	TaN0M0	low	Primary	×
SP22	72	M	TaN0M0	low	Primary	×
SP23	67	M	T1N0M0	low	Primary	×
SP24	39	M	TaN0M0	low	Primary	×
SP25	46	M	TaN0M0	low	Primary	×
SP26	43	F	TaN0M0	low	Primary	×
SP27	28	M	TaN0M0	low	Primary	×
SP28	58	M	TaN0M0	low	Primary	×
SP29	50	M	TaN0M0	low	Primary	×
SP30	42	M	TaN0M0	low	Primary	×
SP31	59	M	TaN0M0	low	Primary	×
SP32	56	M	TaN0M0	low	Primary	×
SP33	38	M	T1N0M0	low	Primary	×
SP34	57	M	TaN0M0	low	Primary	×
SP35	73	M	TaN0M0	low	Primary	×
SP36	69	M	TaN0M0	low	Primary	×
SP37	45	F	T1N0M0	low	Primary	×
SP38	56	M	TaN0M0	low	Primary	×
SP39	54	M	TaN0M0	low	Primary	×
SP40	76	M	TaN0M0	low	Primary	×

* SR, supplementary relapsed tumor. SP, supplementary primary tumor. The TNM cancer staging system was designed to gauge the extent of cancer in a patient's body. T describes the size of the tumor and whether it has invaded nearby tissue, N describes regional lymph nodes that are involved, and M describes distant metastasis (spread of cancer from one body part to another).

Supplementary Table 7. PCR primer sequences for selected genes.

Gene	Forward primer	Reverse primer	RT-PCR
ACTB	GTCACCAACTGGGACGACAT	AGGGATAGCACAGCCTGGAT	RT-PCR
MLL	AAAGCCCTCGAAGGATTAAG	AGCACTGACAACAGGCATGAT	RT-PCR
EP400	GAAGCACAGTAGAGACGGACC	CTGGAAAACTACCCCTTGGTG	RT-PCR
PRDM2	GGGCCATTTGTTGGTGATAAGA	AAGCCCAATTCACATATCGCA	RT-PCR
TMPRSS13	GGCTGCGTGAGGTTTGACT	TCCGGTGAGCACTCTCGAA	RT-PCR
GATA4	GTGTCCCAGACGTTCTCAGTC	GGGAGACGCATAGCCTTGT	RT-PCR
GATA6	CTCAGTTCCTACGCTTCGCAT	GTCGAGGTCAGTGAACAGCA	RT-PCR
MMP2	GATACCCCTTTGACGGTAAGGA	CCTTCTCCCAAGGTCCATAGC	RT-PCR
MMP13	CCAGACTTCACGATGGCATTG	GGCATCTCCTCCATAATTTGGC	RT-PCR
ETS1	TACACAGGCAGTGGACCAATC	CCCCGCTGTCTTGTGGATG	RT-PCR
HOXA9	GTCCAAGGCGACGGTGTTT	CCGACAGCGGTTCAGGTTTA	RT-PCR
GATA4	CTGGGTAGGGGCTGGAGTAG	CTGGCCGAGAGCAGTACG	CHIP
ETS1	CTGGGCTCGGTGAGGAACGCCCT	AGTCCGCGCGAAGTTGGCACTT	CHIP
MLL (g.chr11:118359433)	AAGAAGGGTATGGTTGATTATGTT	AGCTGTAGTTCTATTACCAAGTTT	DNA-PCR
MLL (g.chr11:118375263)	TACCCCCTCCGACAAAAATTTACT	GTGATGGTTACTCTCTTCTCCCCT	DNA-PCR
MLL (g.chr11:118359433)	GTTTATGGATGAGGTTTTGACTCC	ATGAGGTATTATTGGGGCTTGGTT	DNA-PCR
MLL (g.chr11:118359433)	GAGTACCAATTAAAACCAGGTTTG	TCCTGATGTATCTCTGCAACAAAC	DNA-PCR
